# Doped Electrospinned Material-Guides High Efficiency Regional Bone Regeneration

**DOI:** 10.3390/polym15071726

**Published:** 2023-03-30

**Authors:** Manuel Toledano, Cristina Vallecillo, María-Angeles Serrera-Figallo, Marta Vallecillo-Rivas, Aida Gutierrez-Corrales, Christopher D. Lynch, Manuel Toledano-Osorio

**Affiliations:** 1 Faculty of Dentistry, Colegio Máximo de Cartuja s/n, University of Granada, 18071 Granada, Spain; 2 Biosanitary Research Institute, Biosanitary Research Institute, 18012 Granada, Spain; 3 Faculty of Dentistry, Oral Surgery Section, University of Sevilla, Avicena s/n, 41009 Sevilla, Spain; 4 Restorative Dentistry, University Dental School & Hospital, University College Cork, Wilton, T12 E8YV Cork, Ireland

**Keywords:** zinc, doxycycline, silica, regeneration, bone cells, polymer, membrane, vascularization, macrophage

## Abstract

The main target of bone tissue engineering is to design biomaterials that support bone regeneration and vascularization. Nanostructured membranes of (MMA)1-co-(HEMA)1/(MA)3-co-(HEA)2 loaded with 5% wt of SiO_2_-nanoparticles (Si-M) were doped with zinc (Zn-Si-M) or doxycycline (Dox-Si-M). Critical bone defects were effectuated on six New Zealand-bred rabbit skulls and then they were covered with the membranes. After six weeks, a histological analysis (toluidine blue technique) was employed to determine bone cell population as osteoblasts, osteoclasts, osteocytes, M1 and M2 macrophages and vasculature. Membranes covering the bone defect determined a higher count of bone cells and blood vessels than in the sham group at the top regions of the defect. Pro-inflammatory M1 appeared in a higher number in the top regions than in the bottom regions, when Si-M and Dox-Si-M were used. Samples treated with Dox-Si-M showed a higher amount of anti-inflammatory and pro-regenerative M2 macrophages. The M1/M2 ratio obtained its lowest value in the absence of membranes. On the top regions, osteoblasts were more abundant when using Si-M and Zn-Si-M. Osteoclasts were equally distributed at the central and lateral regions. The sham group and samples treated with Zn-Si-M attained a higher number of osteocytes at the top regions. A preferential osteoconductive, osteoinductive and angiogenic clinical environment was created in the vicinity of the membrane placed on critical bone defects.

## 1. Introduction

Bone regeneration processes are still based on the use of membranes whose main function is to perform an occlusive function [1]. This is commonly known as the principle of epithelial exclusion and it is what has guided bone regeneration (GBR) [2]. Currently, there is a growing interest in the development of membranes that actively participate in the healing and regeneration process through interaction with cells and drug release, away from being just a passive barrier [1,3]. Barrier membranes are essential for the establishment of conditions for GBR [4,5]. To enhance hydrophilicity, cell–membrane interactions, mechanical properties and osteogenesis and to confer antibacterial properties, novel composite membranes based on the electrospin of a mixture of (MMA)_1_-co-(HEMA)_1_ and (MA)_3_-co-(HEA)_2_ doped with silicon dioxide nanoparticles (SiO_2_-NPs) are proposed. The composite membranes, in the present research, are loaded with silica nanoparticles to promote their integration with human tissues by inducing scaffold bioactivity and osteoconductivity [6] and by facilitating surface formation of calcium phosphate deposits [7]. Vascularization is a key event for bone remodeling and regeneration to occur, since the skeleton depends on the vascular system to maintain its metabolic balance [8,9]. It is widely accepted that vascular development always precedes osteogenesis [10]. The processes of vascularization and bone formation occur jointly and combined, making it impossible for ossification to happen without the presence of vascularization. Cells must be at a distance of 100 to 200 µm from blood vessels to survive and not affect tissue homeostasis and consequently the regeneration process [11]. During bone regeneration, the presence of osteoprogenitor cells and osteoclasts in the defect site supervenes vascular invasion [11]. Bone materials have been developed promoting their angiogenic and osteogenic activities to accelerate the formation of a vascular network in the defect as a strategy to achieve material-guides high-efficiency bone regeneration [8]. Thus, immunomodulation manipulation has become a valuable instrument to guide the formation of new bone. Therefore, it seems critical in GBR to focus on materials that act as scaffolds of the blood vessels allowing a vascular network that precedes temporally the formation of new bone. Vascularization appears as an essential component of a metabolically active bone regeneration. However, this fundamental principle is frequently neglected in bone tissue engineering studies [10].

Osteoimmunology focuses on the interaction between immune cells and the skeletal system [12]. Bone cells, such as osteoblasts, osteoclasts, osteocytes and immune cells like macrophages share the same micro-environment and a variety of molecules, working together within the concept of osteoimmunology [13]. Bone homeostasis is highly regulated with coordinated crosstalk among these cells [14]. Osteoblasts are bone-forming cells derived from multipotent mesenchymal precursors. These cells produce extracellular proteins, among which osteocalcin, alkaline phosphatase and type I collagen stand out, the latter of which constitutes more than 90% of the bone matrix protein [15]. Osteoclasts come from hematopoietic stem cells; they are specific macrophages in charge of bone resorption. They participate in bone remodeling, dissolving collagen and other matrix proteins during the removal of old or damaged bone, which is then replaced by new bone produced by osteoblasts [15]. Traditionally, bone remodeling has been suggested to be a sequential stepwise process, with initial osteoclast-mediated bone resorption followed by osteoblast-mediated bone formation. However, in certain pathologic skeletal disorders, such as inflammatory bone loss, both processes do not occur consecutively [15]. Osteocytes also play a key role in the formation and remodeling of bone [16]. They are mechano-transducer cells and crucial determinants of bone quality [16].

Once the biomaterials are implanted into the host tissue, one of the first cell types they interact with are cells derived from the monocyte/macrophage lineage. Immune response is also critical in bone healing. Macrophage is a dynamic cell, derives from monocytes and participates in induction and resolution of metabolic/inflammation purposes [13]. Macrophages play pivotal and dynamic roles in bone regeneration, as monocyte migrates from the circulation to the local tissue. Monocyte differentiates into macrophage, and depending on the stimulus and microenvironment, polarizes into different types in vivo, including non-activated M0, classical or pro-inflammatory M1 and anti-inflammatory and pro-regenerative M2 [13]. Skeletal or osteal macrophages (osteomacs) have been reported to contribute to bone homeostasis and regeneration and to take part in an essential function, as remodeling [17]. They could even become osteoclasts or multinucleated giant cells, both related to the rejection of implanted biomaterials [18]. Most of the M1 macrophages are associated with the resistance towards infection. M2 is directly related to tissue remodeling, repair and wound healing. Converting the macrophage phenotype from pro-inflammatory to anti-inflammatory by modulating cytokines and signaling pathways, eventually altering its function and its capability to regulate the direction of stem cell differentiation and promote osteogenesis [13]. It was hypothesized that it would be possible to modulate the ratio of macrophage phenotypes (M1/M2) by altering the chemical composition of the membranes. Investigating and understanding, in depth, the mechanisms underlying macrophage polarization, and what the switch between M1 and M2 states depends on is of great interest and could contribute to new therapeutic approaches [19]. Factors such as macrophage morphology and shape influence the modulation of macrophage phenotypic polarization [20,21]. Pro-inflammatory M1 and anti-inflammatory M2 macrophages denote two poles of a *continuum* of overlapping cellular activities. Morphological parameters were used to define the polarization outcomes, as previously reported [22]. M1 were nearly round or irregularly spherical with more lamellipodia [20]. M2 showed elongated shape and long filopodia [20].

Due to the above, it is of interest to study the effects of the membrane that covers a defect, based on the vascularization represented by the presence of blood vessels, the osteomodulation produced by macrophages and the osteogenesis caused by bone growth cells. Our objective is to assess the regional vascular vessel distribution and cell population in critical-sized defects of calvarial bone in a rabbit model using novel nanostructured silica-loaded membranes doped with zinc or doxycycline. The null hypothesis to be proved is that the silica-based novel membranes doped with zinc and doxycycline do not facilitate similar vascular vessel distribution and cell population in the distinct regions of the defect.

## 2. Materials and Methods

### 2.1. Membrane Functionalization

Nanostructured membranes were manufactured by NanomyP^®^ (Granada, Spain). The membranes were made using an innovative polymeric blend (PolymBlend^®^): (MMA)_1_-co-(HEMA)_1_/ (MA)_3_-co-(HEA)_2_ 50/50 wt, doped with 5%wt of SiO_2_-NPs. Polymers have an average molecular weight of 200 and 2000 kDa, respectively. After electrospinning (Anex S1), membranes were immersed in a sodium carbonate buffer solution (333 mM; pH = 12.5) for 2 h; the membranes were then activated with carboxyl groups, forming HOOC-Si-Membranes. I was produced due to ester bond partial hydrolysis [23]. Subsequently, the membranes were rinsed with distilled water and dried in a vacuum oven [23,24]. Immediately after, using carboxyl groups to complex divalent cations, nanostructured membranes were functionalized with zinc. Doxycycline (Dox) was fixed on the membranes by acid–base interactions between amino groups of Dox and membranes’ carboxyl groups. Aiming to achieve this, HOOC-Si-Membranes were immersed under continuous stirring at room temperature and in aqueous solutions (pH = 7) of both 330 mg L^−1^ of ZnCl_2_ and 800 mg L^−1^ of Dox [25]. Afterwards, membranes were washed, dried in a vacuum oven and sterilized using an ultraviolet radiation sterilization desk (J.P. Selecta, Barcelona, Spain. Three different membranes were designed: (1) SiO_2_-NP doped membrane, HOOC-Si-Membrane (Si-M), (2) SiO_2_-NPs doped membrane functionalized with Zn, Zn- HOOC-Si-Membrane (Zn-Si-M) and (3) SiO_2_-NP doped membrane functionalized with Dox, Dox-HOOC-Si-Membrane (Dox-Si-M). These membranes have been previously characterized using atomic force microscopy (AFM), field emission scanning electron microscopy (FESEM) surface characterization, acellular static in vitro bioactivity test, nanomechanical property assessments and cell viability analysis [24,26]. Moreover, cell proliferation, differentiation and gene expression were tested using osteoblasts from the human MG63 osteosarcoma cell line [24,27,28].

### 2.2. Animal Experimentation

Six New Zealand breed were selected for this research. The experimentation white rabbits presented identical characteristics (weight 3.5–4 kg, age 6 months). Rabbits were adequately sheltered daily ad libitum; food and water was provided by Rabbit maintenance Harlan-Teckland Lab Animal Diets (2030). This experiment was carried out in accordance with the US National Institutes of Health (NIH for Care and Use of Laboratory Animals) and European Directive 86/609/EEC guidelines concerning animal care and use for experimentation. It also complied with the European Directive 2010/63/EU about animal protection for scientific purposes and all local laws and regulations. The minimum number of animals, for ethical reasons, as required by the legislative framework, was used. In addition, the Ethics Committee of the Institution (CCMI-Ref026/18) approval was obtained. Concerning animal experimentation and histological methods, comparable models have been published [26,29].

This investigation reports the results from a subset analysis of the specimens whose histological results are described in a separate publication (Toledano et al., 2020 [7]).

### 2.3. Surgical Procedure

Animals’ vital signs were obtained before starting the surgery. After checking vital signs, they were immobilized and anesthetized using both Propofol (5 mg/kg) and Midazolam (0.25 mg/kg) infiltrating them intravenously for induction along with 2.8% inspired sevoflurane gas inhalation. Ketorolac (1.5 mg/kg) and tramadol (3 mg/kg) provided the analgesia. Once animals were sedated and prepared, incisions between their eyes and their ear bases, with Number 15 scalpel blade, were made. The three incisions formed a triangular field made by connecting the two incisions with another in the midline of the skull. The bone surface was exposed after the connective, muscular and epithelial tissue removal from the operation field, using a Prichard periosteotome. Once the skull surface was exposed and washed with sterile saline solution, four critical bone defects were made on the parietal bone (Figures S1 and S2). Piezo surgery was handled to remove the inner table and the medullary bone in every defect, permanently controlling the depth with a periodontal probe. Three bone defects were covered by randomly allocated membranes. The fourth bone defect was not covered (sham group).

A specific software (Research Randomizer, V. 4.0, Urbaniak GC and Plous S, 2013) was used to generate randomization, Tissucol (Baxter, Hyland S. A. Immuno, Rochester, MI, USA). A fibrin tissue adhesive was employed to immobilize the membranes, and it was applied on the bone rims adjacent to the defects. The flaps were repositioned, confirming no mobility and proper adhesion of the studied membranes. Sutures were undertaken (Figure S3). In order to clean the wound, a sterile saline solution was applied. Carprofen 1 mL/12.5 kg and buprenorphine (0.05 mg/kg) was administered for anti-inflammatory analgesia. Six weeks after surgery, the animals were sacrificed with a potassium chloride solution intravenous overdose. After the procedure, the obtained tissue samples were cut and individually separated [6].

### 2.4. Histology

**Figure 1:**
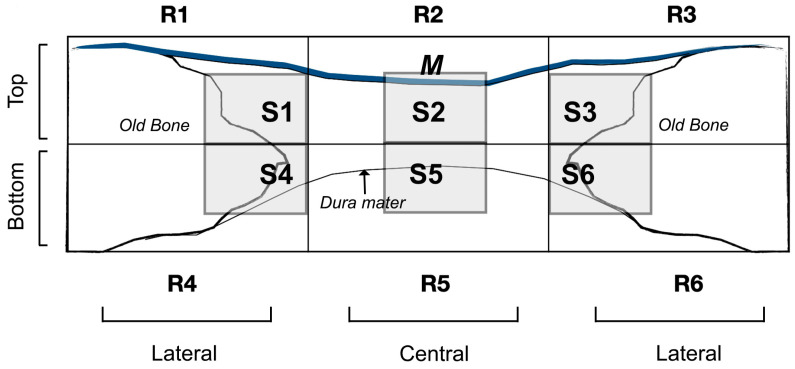
Schematic diagrams showing the defect and the area of measurements for histology. A software rectangular grid consisting of six regions covered the entire area of the defect. Top (close to the membrane (M) area) (R1, R2 and R3) and bottom (close to the *dura mater*) (R4, R5, R6) areas are also represented. Lateral or peripheral (adjacent zone to the old bone or native calvarial) (R1, R3, R4 and R6) and central (sum of the remaining areas) (R2 and R5) zones are also represented. Six sections (S1 to S6) for analysis within each region were segmented.

From each rabbit skull, samples were prepared, cutting them in the anatomical sagittal plane, and fixing the undecalcified bone with a 5% buffered formaldehyde solution (pH 7.4). Blocks from the regenerated bone defect were retrieved using an oscillating autopsy saw (Exakt, Kulzer, Wehrheim, Germany). Then, the dissected specimens were immersed in 4% formaldehyde and 1% calcium solution, included in acrylic resin and prepared for ground sectioning. All sections were coded and a light microscope (Nikon Tokyo, Japan) was used to evaluate blindly histology. For rapid contrast tissue analysis, a metachromatic dye and histological staining (Merck Toluidine Blue-Merck, Darmstadt, Germany) were employed with a 1% toluidine blue (TB) solution (pH of 3.6) adjusted with HCl. Subsequently, samples were exposed to the dye with distilled water, and air dried during 10 min at room temperature (23.0 ± 1.0 °C). An Eclipse LV100 microscope (Nikon, Tokyo, Japan) with 20× and 50× lenses was employed to visualize bone cell population and vasculature from toluidine staining studies. A DSPDS-Fi1 camera (Nikon, Tokyo, Japan) along with NIS Elements BR 4.0 software (Nikon, Tokyo, Japan) were used to take the pictures. In each defect, blood vessels, osteocytes, osteoblasts, osteoclasts and macrophages (M1 and M2) were assessed at the toluidine blue images. The M1 and M2 macrophage number and the ratio M1/M2 were analyzed with morphology criteria by coloration with toluidine blue. They exhibited a distinctive morphology, with round, vacuolized or fried egg-shaped [30] for M1, or elongated, spindle-shape or fibroblast-like appearance for M2 [22]. Image analyses were realized using ImageJ software. The measurements were assessed on the total defect area level and by dividing this area into regions that constitute top and bottom regions or central and lateral regions. One section (600 × 400 micrometer) for measurement within each area was selected as representative of each area; therefore, six sections (S1–S6) were considered for analysis (Figure 1). In the regions located on the edges of the defect (R1, R3, R4 and R6) the chosen section was the closest to the neighboring regions, that is, to the most central and upper/lower region. This protocol permitted not only handling in the most reproducible way to locate the sections, but also finding new cellularity. This also allowed avoiding the already formed old bone that is usually found in the areas located less close to the created defect. The sections of the central regions were chosen at the central level of the defect in order to appreciate the true effect of the membrane in the central area of the defect. In each bone defect, four images were taken and analyzed.

### 2.5. Statistical Analysis

Means and standard deviations (SDs) were obtained. Non-parametrical Friedman test was used for variance analysis and non-parametric pairwise comparison of Friedman rank sums method for post-hoc analysis was employed. Level of significance was set at *p* ≤ 0.05. Assessment was undertaken by means of IBM SPSS Statistics v.24 software package.

## 3. Results

### 3.1. Blood Vessel Assessment

**Figure 2:**
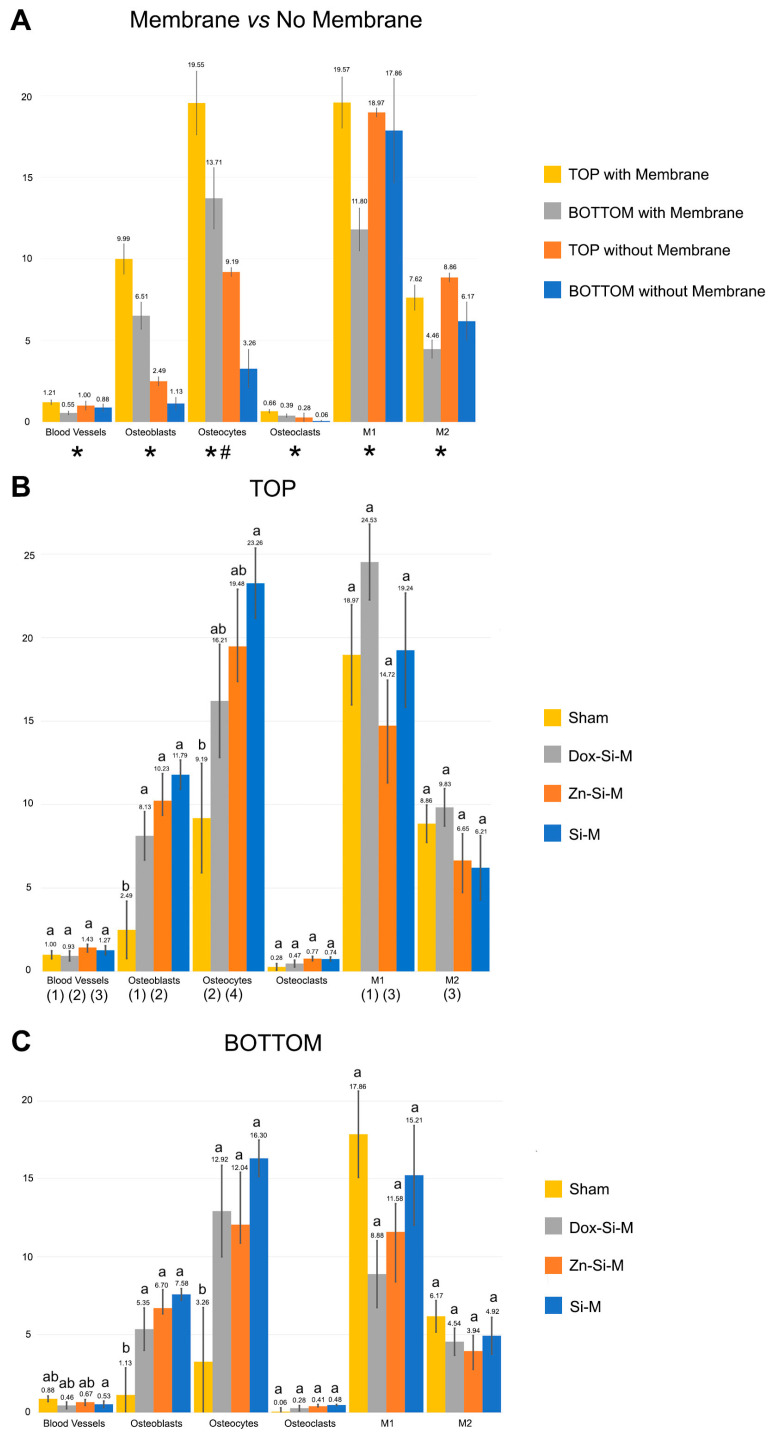
Mean ± Standard Error of blood vessels and bone cell counts at the selected study section when comparing; (**A**) the presence of membrane over the defect versus no membrane and (**B**) top (R1, R2, R3) and (**C**) bottom (R4, R5, R6) regions. Statistical results (*p* values) after pairwise comparisons. Similar lower-case letters indicate no differences between membranes within the same cell group. Asterisk means significance (*p* < 0.05) after comparing top versus bottom in the presence of membrane over the defect. Hash means significance (*p* < 0.05) after comparing top versus bottom without membrane over the defect. (1) means significance (*p* < 0.05) after comparing top versus bottom with Si-M. (2) means significance (*p* < 0.05) after comparing top versus bottom with Zn-Si-M. (3) means significance (*p* < 0.05) after comparing top versus bottom with Dox-Si-M. (4) means significance (*p* < 0.05) after comparing top versus bottom in sham group. The numerical data are collected in Supplementary Materials Table S1.

**Figure 3:**
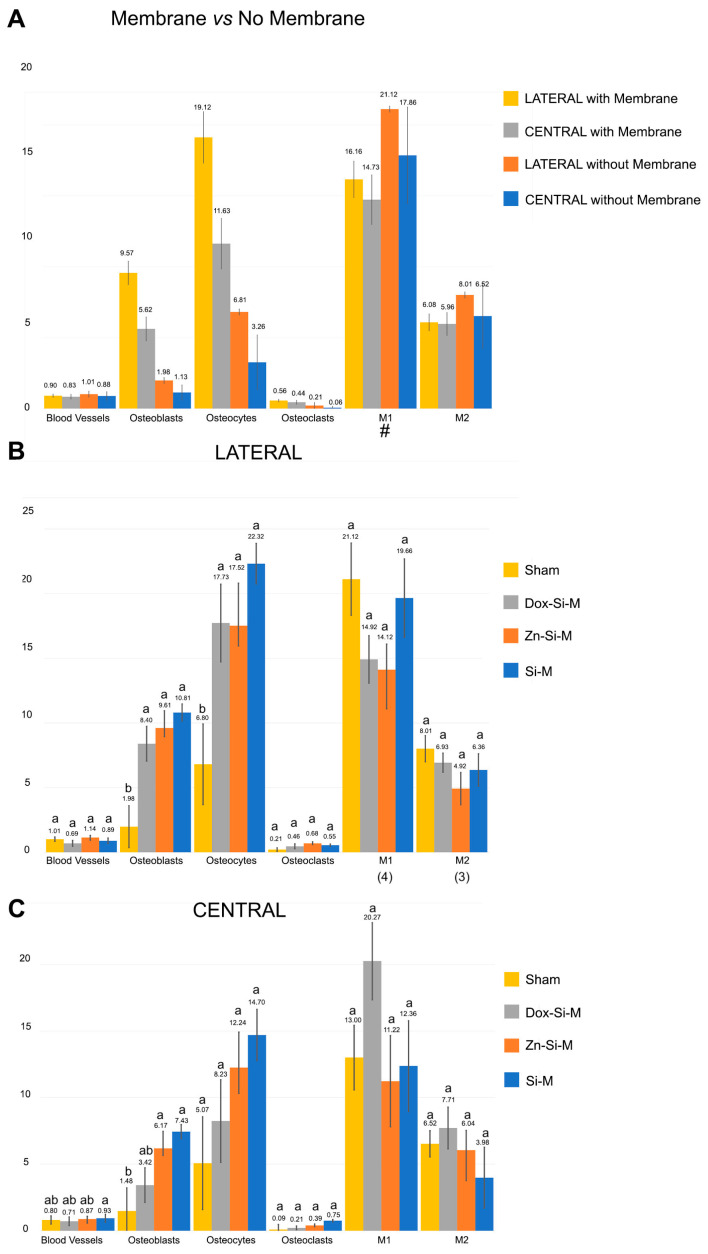
Mean ± Standard Error of blood vessels and bone cells counts at the selected study section when comparing (**A**) the presence of membrane over the defect versus no membrane and (**B**) lateral (R1, R3, R4, R6) and (**C**) central (R2, R5) regions. Statistical results (*p* values) after pairwise comparisons. Similar lower-case letters indicate no differences between membranes within the same cell group. Hash means significance (*p* < 0.05) after comparing lateral versus central without membrane over the defect. (3) means significance (*p* < 0.05) after comparing lateral versus central with Dox-Si-M. (4) means significance (*p* < 0.05) after comparing lateral versus central in sham group. The numerical data are collected in Supplementary Material Table S2.

**Figure 4:**
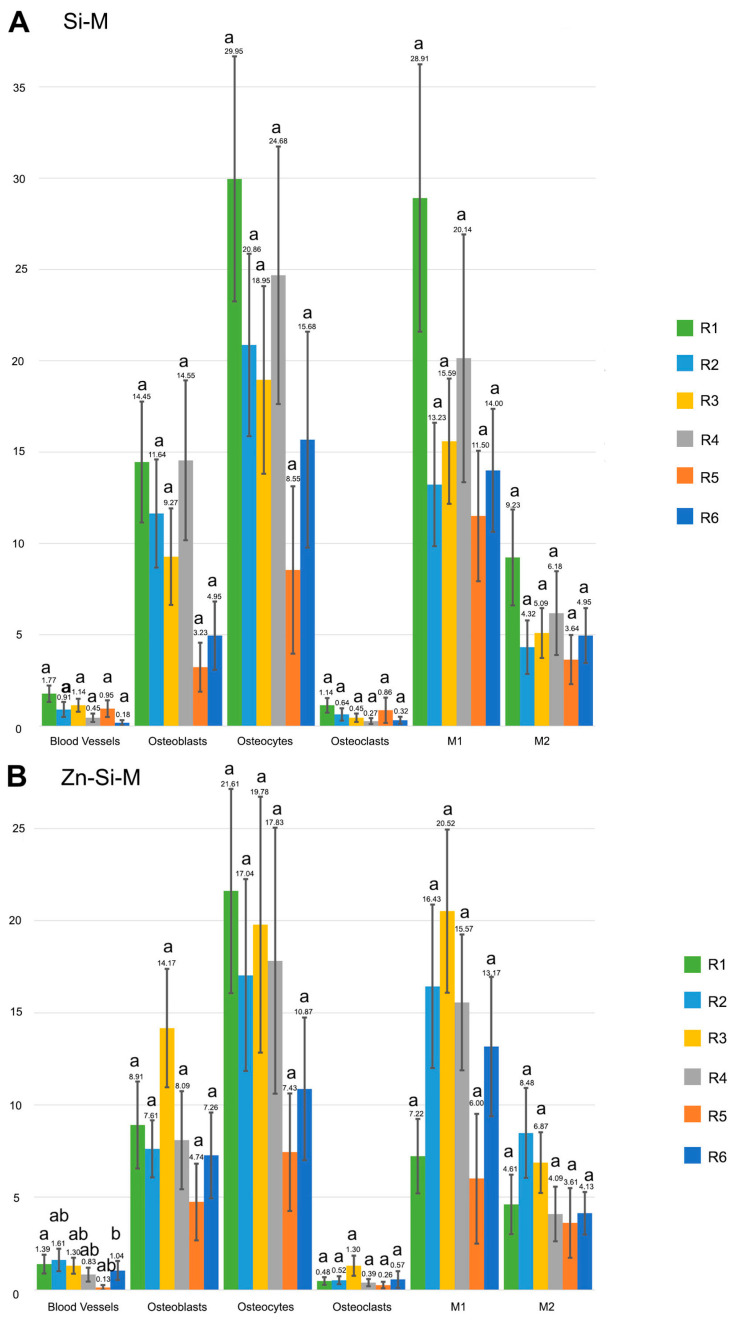

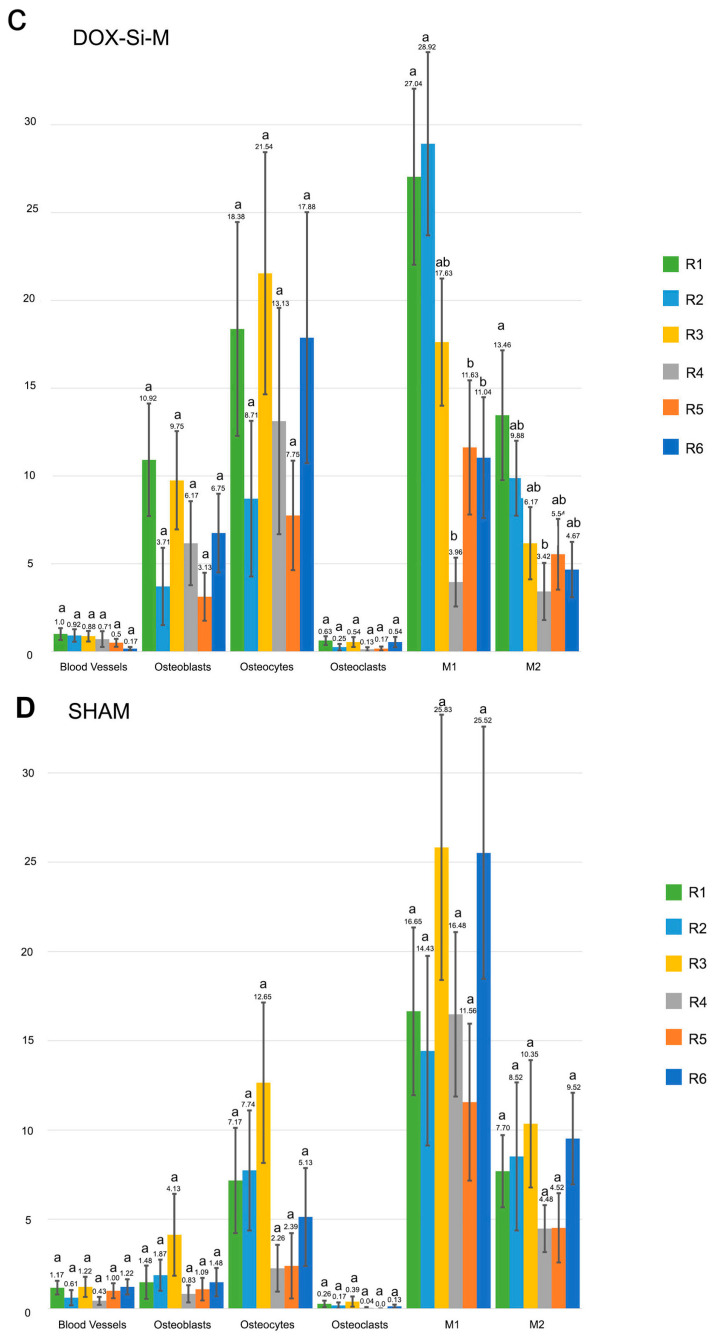

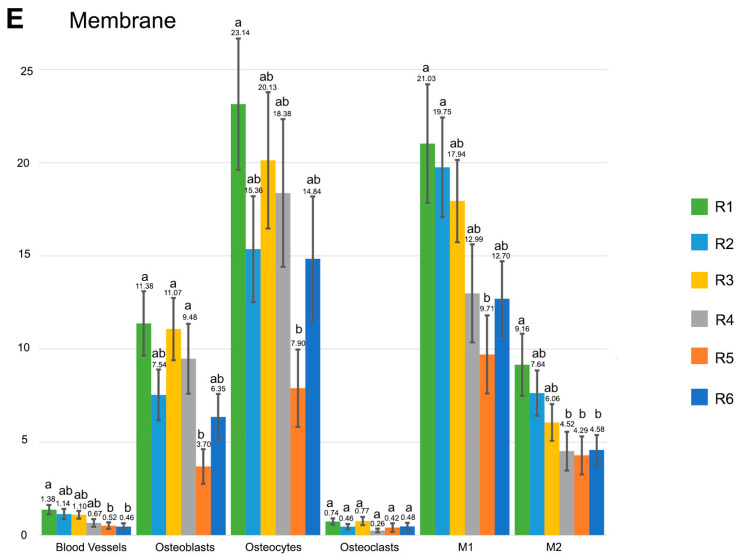
Mean ± Standard Error of blood vessels and bone cells counts at the selected study section within each of the regions into which the defect is divided in (**A**) Si-M, (**B**) Zn-Si-M, (**C**) Dox-Si-M, (**D**) sham group and (**E**) with the presence of membrane. Similar lower-case letters indicate no differences between regions within the same cell group. The numerical data are collected in Supplementary Materials Table S3.

Blood vessels were more abundant in the top regions of the defect than in the bottom, regardless the type of membrane (Figure 2A,B). When the uncovered defects were analyzed, the presence of blood vessels was similar in all regions of the defect (Figure 2A and Figure 3A). When the defect was covered with membrane, the growth of blood vessels increased significantly in the top regions. All membranes obtained a significantly higher count of blood vessels in the top regions compared to the bottom ones. Zn-Si-M achieved the highest number of blood vessels both in the top/bottom and lateral/central regions (Figure 2B and Figure 3B). Regardless of whether or not a membrane was present, when vasculature was analyzed by region, only Si-M showed significant differences when blood vessels were assessed. The highest count of blood vessels was found in R1 (top left) of the bone defect, and the lowest in R6 (bottom left) (Figure 4). In the top central zone, R2, where there is more influence from the membrane and less from the edges of the defect, Zn-Si-M reached the highest blood vessel counts (Figure 4).

**Figure 5:**
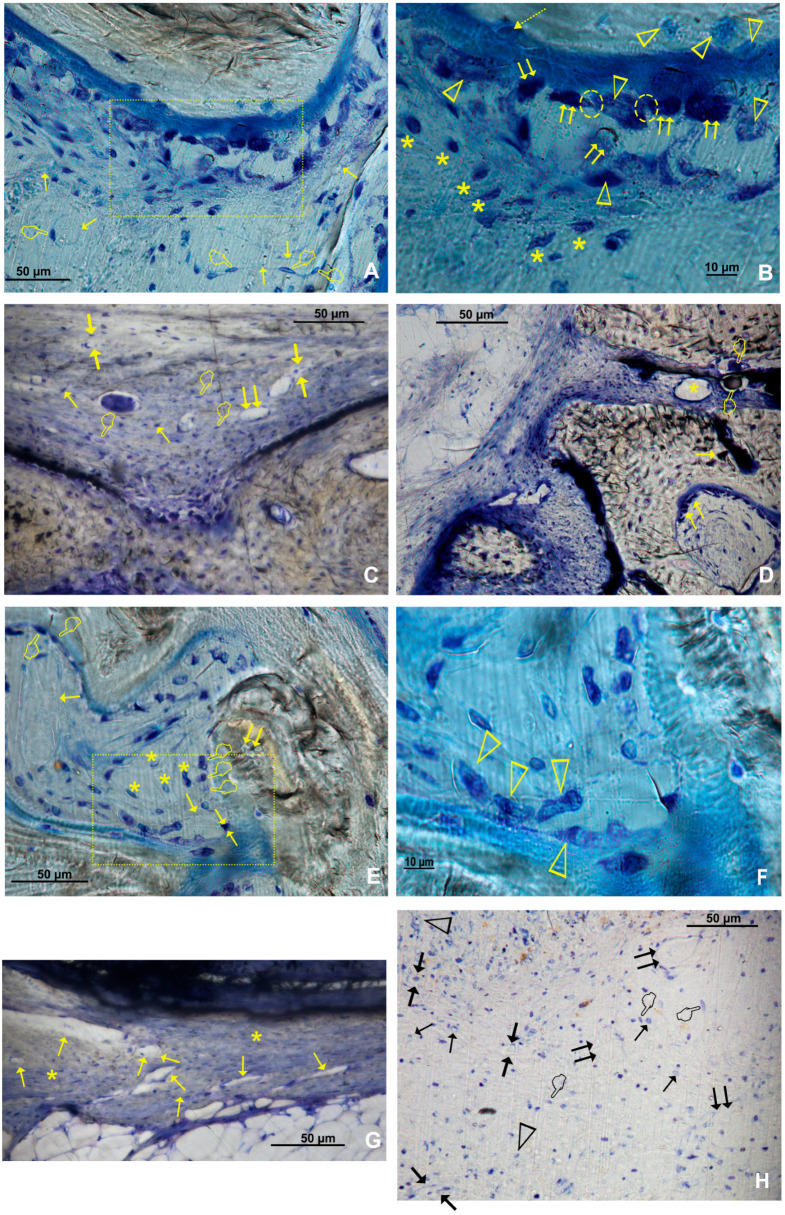
(**A**) Bone histology image obtained after using Zn-Si-M, at R1 region, by dye with toluidine blue to visualize bone cells and vascular vessels, at six weeks of healing time. The single arrows indicate the presence of vascular vessels. Endothelial cells are indicated by pointers. The dotted square, magnified, indicates Figure 2B. (**B**) Bone cells in a procedure of bone remodeling. Both osteoblasts (double arrows) and osteoclasts (arrow heads) are in close contact with marrow elements. Some entrapped osteoblasts (dotted arrow) may be observed by the osteoid bone. Interconnected contact cells (osteoblast–osteoclast) or cross-talk and extracellular matrix interactions are reflected by dotted circles. Asterisks indicate some macrophages. (**C**) Bone histology images obtained after analyzing the R2 section when Dox-Si-M was used; M1 (pointers) and M2 (single arrows) macrophages were observable. Big (double arrows) and small blood vessels (faced arrows) are also shown. (**D**) Bone histology images obtained after analyzing the R3 sections in samples treated with Dox-Si-Ms. Single arrows indicate the presence of osteoblasts, with typical cuboid shape. Pointers indicate osteoclasts. Asterisks indicate a vascular vessel. (**E**) Bone histology images obtained after using Si-M at R1 regions, by dye with toluidine blue to visualize blood vessels (single arrows) at six weeks of healing time. Monocytes attached to the endothelial layer and adhered to the vascular vessels, forming part of their walls (arrow heads). A canopy of macrophages and osteomacs (asterisks) may be noticed. Osteocyte with its mineral lacuna (double arrow), osteoblast (faced arrows) and osteoclast (pointers) may be observed. The dotted section in (**A**) is magnified in (**F**). (**G**) Bone histology images obtained after using no membrane at R1 region, by dye with toluidine blue to visualize blood vessels, at six weeks of healing time. Single arrows indicate the presence of blood vessels. Bone cells are in close contact with marrow elements and the contiguous vasculature; macrophages (asterisks) may also be observed. (**H**) Bone histology images obtained after analyzing the R2 section in the control group (no membrane), by dye with toluidine blue to visualize macrophages at the bone defect (**D**), at 6 weeks of healing time. M1 (pointers) and M2 (single arrows) macrophages were observable. Big (double arrows) and small blood vessels (faced arrows) are also shown. Some monocytes were indicated (arrowheads).

Histological assessment showed multiple blood vessels and bone cells in close contact throughout the bone defect (Figure 5, Figures S4 and S5).

### 3.2. Osteoblast Assessment

The number of osteoblasts when using Si-M and Zn-Si-M was higher in the top regions than in the bottom regions of the defect (*p* < 0.05) (Figure 2B). When the membranes were present and cellularity was analyzed by region, the highest count of osteoblasts was found in R1, R2 and R4 of the bone defect (Figure 4), and the lowest at R5, but without significant differences (Figure 4B and Figure 5). Bone defects performed in animals treated with Si-M showed the highest counting of osteoblasts (Figure 4). When top and bottom regions of the defect were analyzed, osteoblasts in the control group were present in the lowest amount (Figure 4C). Similar performance occurred when lateral and central areas were analyzed (Figure 4D), but without significant differences. Osteoblasts showed a strongly basophilic cytoplasm, indicating the ability to produce a large amount of extracellular matrix (Figure S6), i.e., the osteoid bone that was observed as a homogeneous fringe in a clear blue color between the aligned osteoblasts and the mature bone (Figure 5, Figures S4 and S6).

### 3.3. Osteoclast Assessment

The number of osteoclasts was higher at the top than at the bottom of the defect in the presence of membrane. The distribution of osteoclasts was similar without membrane (*p* < 0.05) (Figure 2A). The distribution of osteoclasts was alike at the central and lateral regions of the defect in all groups and was not influenced by the presence of membranes (*p* > 0.05) (Figure 3A,B). Osteoclasts reproduced a fitted junction between the bone surface and the basal membrane of the cell, formed as a sealed compartment, known as the acting ring, which includes the characteristic ruffled border as a part of an external vacuole. As opposed to the contact surface of the bone through this ruffled border forming a sealing zone (podosome), the functional secretory domain can be noticed (Figure 5, Figures S4 and S6). Both osteoblasts and osteoclasts appeared in close contact with marrow elements and the contiguous vasculature (dotted circles in Figure 5D and Figure S6B,C).

### 3.4. Osteocytes

The calvarial defects in both the sham group and those treated with Zn-Si-M attained a higher number (or counting) of osteocytes at the top regions of the defect than defects treated with Si-M or Dox-Si-M (Figure 2B). In general, osteocytes showed a higher counting at the top areas of the defects, regardless of the presence or not of a membrane (Figure 2A). Osteocytes were more concentrated at the R1 region of the defect (Figure 4), and region R5 showed the lowest amount of osteocytes, but without significant differences (Figure 4B). In the control group, the top, bottom and lateral areas of the defect attained the lowest amount of osteocytes within the bone defect (Figure 4C,D). Histological images show the presence of osteocytes and canaliculi crossing over the bone matrix, and their characteristic dendritic matrix morphology joining at the gap junctions (Figure 5, Figures S5, S6 and S8).

### 3.5. M1, M2, M1/M2 Assessment

In general, the presence of membranes covering the bone defect influenced a higher count of bone cells and blood vessels than in the sham group at the top regions of the defect (Figure 2A). When top and bottom regions of the defect were compared in the present research, the obtained histological images allowed one to detect that pro-inflammatory M1 appeared in a higher number in the top regions than in the bottom regions of the defect when Si-M and Dox-Si-M were used (Figure 2B).

M1 and M2 were more abundant at the top regions of the defect when the membranes were present (Figure 2A) and ended up equally distributed throughout the bone defect when lateral and central areas were analyzed in the presence of membranes (Figure 3A, Figure 5 and Figure S9).

Only samples treated with Dox-Si-M showed a higher amount of anti-inflammatory and pro-regenerative M2 at the top regions of the defect than in the bottom ones (*p* < 0.05) (Figure 2B, Figure 5C and Figure S7).

Concerning cell population, pro-inflammatory M1 macrophages appeared in a higher number in the lateral than in the central regions of the defect when the sham group was analyzed. Differences did not appear in the rest of the groups (Figure 3B). The amount of anti-inflammatory and pro-regenerative M2 macrophages was higher at the lateral regions when defects were treated with Dox-Si-M (Figure 3B). The presence or absence of membranes covering the bone defects influenced neither the bone cell count nor the blood vessel count when lateral vs central regions were compared (Figure 3A). The M1 count was statistically different in the R1 region, with the animals being treated with Si-M, which showed the highest number of M1, and those treated with Zn-Si-M attained the lowest number of M1 (Figure 4). The M1/M2 ratio obtained its lowest value in the groups where the absence of membranes (control) was assessed (Table 1). Significant differences in M1/M2 ratio were obtained at the top regions of the bone defect, with the group of animals treated with Dox-Si-M attaining the highest average (6.08) (Table 1). The control group attained the lowest M1/M2 values in the central regions of the defect (1.69) (Table 1). Histology images show M1 (single arrows) and M2 (pointers) macrophages, observable at different regions of the defect (Figure 5, Figures S9 and S7). M1 showed increase size and granularity, nearly round or irregularly spherical shape, with more lamellipodia. M2 exhibited elongated or spindle-shaped appearance and long filopodia, with a loss of nuclear volume and induction of chromatin condensation (Figure 5, Figures S4 and S9).

## 4. Discussion

Vascularization is crucial for an osteoconductive and osteoinductive clinical environment that will determine the success in the treatment of therapies related with bone regeneration. Bone reconstruction, preceded and accompanied by angiogenesis, is a well-orchestrated process consisting of three main successive phases, inflammation, repair and remodeling, where osteoblasts, osteoclasts and macrophages participate in concert [17]. For this process to take place, the formation of blood vessels is an essential condition. The presence of membrane at the defect lead to not only a higher count of vascular vessels, but a count of all cell populations at the top region of the bone defect (*p* ≤ 0.05) (Figure 2A).

Bone is a highly vascularized tissue with the ability to remodel and repair itself [31]. Angiogenesis always precedes osteogenesis [31]. In this research, vascular vessels were significantly more present at the top regions of the bone defect when any type of membrane was placed (Figure 2A,B), appearing in close vicinity to cell groups (Figure 5, Figures S4 and S8). The presence of membrane over calvaria defect provided a vascular neoformation close to 150% in the top regions R1, R2 and R3 (Figure 2A,B and Figure 4). The exposed experimental findings suggest that silica-loaded membranes, doped or not, may also have an active role in promoting vascularization and osseous regeneration next to the passive barrier function. The placement of a membrane with the proposed composition could have acted as a scaffold for vascularization. Other membranes used in GBR are non-resorbable, such as polytetrafluoroethylene (PTFE), which has the great drawback of low cell adhesiveness, being useless as an active mechanism for cell promotion [29,32,33,34]. With our membranes, due to the porosity and the arrangement of the fibers, a revascularization effect of the defect is achieved. This significant revascularization does not occur when no membrane is there. The active effect of the membrane on vascularity is reflected by a significant increase in the blood vessel count at the top versus the bottom regions (Figure 2A,B). These differences disappear when comparing the central regions with the lateral ones (Figure 3A,B), showing that angiogenesis has occurred mainly in the areas adjacent to the membrane throughout its entire extension over the defect. Udagawa et al., (2012) [31] studied capillary angiogenesis at the bilateral borders of critical and non-critical size defects. The authors concluded that capillary bed formation may be the key to understanding the difference in bone regeneration between size critical and non-critical defects [31]. The formation of the capillary bed and the circulation of blood flow from one side of the defect to the other may be crucial in bone healing and regeneration [31]. Taken together with our results, this could mean that in critical defects, such as those used in this research, placing a membrane has a positive effect, not only because of the compartmental effect, but also because it serves as a scaffold for a vascular network formation from one side to the other of the defect. This can also be verified by observing the different cell groups that play a leading role in bone formation and repairing events, such as osteoblast, osteoclast and osteocytes [35]. These cell groups had a higher amount in the presence of a membrane and especially in the top regions (Figure 4). In the presence of a well-developed vascular network, osteoblasts produce osteoid, calcify and differentiate into osteocytes [10]. Mineralized bone needs to be nourished from vessels; it cannot be nourished by osmosis or diffusion over long distances. Osteocytes cannot survive if they are more than 100 μm away from the nutrient vessels [10]. Therefore, vascular growth determines the cellularity directed to osteogenesis.

The microenvironment that regulates and coordinates the crosstalk among osteoblasts, osteoclasts, osteocytes and vascular vessels may be observed in Figure 5 and Figure S6. Multinucleated cells, which may be either osteoclast or foreign-body giant cells produce VEGF, promoting angiogenesis during bone healing [36]. Clear signs of remodeling, based on both recruitment of cells and the supply of nutrients [37], have been observed (Figures S4 and S6B). Osteoblasts, more abundant at the top regions of the defect in samples treated with Zn-Si-M and Si-M (Figure 2 and Figure 3) secrete VEGFs, and endothelial cells secrete bone morphogenetic protein (BMP)2 to combine osteogenesis and angiogenesis [38]. Bone generation initiates with osteogenic differentiation of mesenchymal stem cells into osteoblasts [14]. Osteoblasts produce mineralization of bones, apart from expressing osteogenic and osteoclastogenic factors [27,39]. The incorporation of silicon, which is present in all tested membranes, has been shown to enhance both bioactivity and osteoblast-like cell activity [40]. It might be inferred that both Si-Ms (Figure 5 and Figure S9A) and Zn-Si-Ms (Figure 5A,B and Figure S9B), which increased osteoblasts at the top region of the defect (Figure 2B), equally stimulate, preferentially at the top regions close to the membrane, the stem cells.

As observed in the present research, osteoclasts were uniformly distributed through the bone defect (Figure 2A,B). Thus, the amount of osteoclasts was lower than the number of osteoblasts in both top/bottom and lateral/central regions of the defect (Figure 2 and Figure 3). Osteoclasts were shown in the vicinity of the membrane toward the bone defect, close to some multinucleated giant cells and M1 macrophages (Figure S4). The formation and presence of these multinucleated giant cells did not hinder bone formation. As such, a classical foreign body reaction may be excluded [41]. Dox suppresses osteoclast activity [42]. A decrease in osteoclasts after treating the bone defect with Dox-doped membranes has been associated with an increase in osteoblasts [7]. A reduction in the number of osteoclasts leads to a lower demineralization, increase bone formation and inhibition of the collagenolitic activity, due to the presence of doxycycline [43], favoring bone remineralization [44].

Zn-Si-M caused a major presence of osteocytes, osteoblasts and vascular vessels in the top regions of the bone defect (Figure 2 and Figure 3), mostly concentrated in the trabecular woven bone that emerge in the vicinity of the membrane (Figure S6). A subpopulation of osteoblasts is terminally differentiated into osteocytes in the process of bone generation [14]. Osteocytes reside in lacuna-canalicular porosities within the mineralized bone matrix (Figure 5, Figures S4B and S5B), forming an organized intercellular network interconnected by dendritic cell processes [45]. The dendritic morphology of osteocytes could have contributed to enlarging the surface area beneath the Zn-Si-M (Figure S6B) [28]. The carboxylate groups might have functioned as cell binding sites, at the proper membrane, probably after Zn^++^ release [46,47]. The generalized increase in both osteoblasts and osteocytes at the top region of the defect (Figure 2B) was probably influenced by the higher loss modulus of these type of membranes that contributed to maintaining their in vitro advanced stability and mechanical performance [7].

Macrophages also play an early role in angiogenesis [17]. Therefore, it could be expected that those regions where there is a greater presence of blood vessels coexist with a higher macrophage count. However, no significant differences were observed in the macrophage count between the presence or absence of membrane, with the count being higher in the sham group (Figure 4A). Macrophages aggregate, produce adenosine triphosphate (ATP) and gradually polarize to the M2 phenotype, secreting vascular endothelial grow factor (VEGF) which stimulates the generation and chemotaxis of endothelial cell precursor cells [17] and also promote osteoblasts differentiation [48]. There is a synergistic crosstalk between the osteoblast precursors and endothelial cells [14], which can be appreciated in our histological analysis, due to their interconnected contact (Figure S8). M1 initiates angiogenesis by secreting VEGF. M2 maintains the stability of the vascular network formation by secreting related growth factors and coordinating the assembly of the extracellular matrix [17,49]. Silica inducted both angiogenesis and osteogenesis via monocytes and macrophage immunomodulation, modulating the proliferation of endothelial cells and the formation of endothelial tissues [42,50]. All the membranes studied contain silica, so this increase in the macrophage count could be attributed to its presence. However, the absence of a membrane control group without silica and the high macrophage count in the sham group prevents us from affirming this relationship. What can be affirmed is the early inflammatory response produced by the immune cells on the surface of the material which determines the fate of the bioimplant in the body even before angiogenesis and osteogenesis [17]. Si-M and Dox-Si-M conditioned a greater presence of pro-inflammatory M1 in the top regions of the defect, in the vicinity of the membrane (Figure 2B, Figure 5C and Figure S9A); therefore, the M1 phenotype was displayed and distinguished by a high pro-inflammatory mediator expression [17]. M1 mainly secrete proinflammatory cytokines such as Tumor necrosis factor-α (TNF-α) and IL-1, IL-6 which are responsible for the recruitment of immune cells at the defect and initiation of the acute inflammatory response, leading to inflammation, tissue injury and fibrosis [51]. IL-10, related with M1 augmentation, plays a central role in tissue repair and neovascularization [13,52], increasing osteogenesis and decreasing osteoclastogenesis via activating exosomal IL-10 mRNA to cells, directly [13,39,53]. Thereby, M1 lay the foundation for subsequent bone tissue repair [17]. An upregulation of several osteogenic markers has been described in the presence of silica-loaded membranes [1], and all membranes used in the present research contain silica in their composition. Si-M and Dox-Si-M have inducted lower M1 counts at the bottom regions of the bone defect in the present animal experimentation (Figure 2 and Figure 3). When the M1 population decreases, the digestive matrix metalloproteases (MMPs) produced by M1 diminish [54]. Dox is an inhibitor of MMPs [43], and thus its pro-healing role was jeopardized [55]. M1 were equally distributed when the bone defects were treated with Dox-Si-M and comparisons were done between central and lateral regions of the defect, though they preferentially concentrated at lateral or peripheral zones (adjacent zone to the old bone or native calvarial: R1, R3, R4 and R6), concretely in R1 regions (Figure 3 and Figure 4).

Macrophages also undergo polarization to M2, in order to stimulate osteogenesis [14]. We speculate that the increase of M1 at the top regions of the defect might have conditioned the correlative raise of anti-inflammatory and pro-regenerative M2 in the same locations when Dox-Si-M was placed on the defect (Figure 2A and Figure 5C). In the event that M2 macrophage polarization is promoted, bone regeneration will occur around the biomaterials. Inflammation seems to act as a switch to turn on the healing phase. Hence, the presence of M1 during the initial inflammation stage is crucial for normal bone healing [17]. M1 that could not switch to the M2 phenotype in time would secret pro-inflammatory cytokines continuously, resulting in delayed healing and chronic inflammation. Premature M2, due to excessive production of fibrotic cytokines, could form a fibrous capsule on the surface of the material, negatively affecting membrane integration [17]. Dox can induce an M1 to M2 subtype polarization [30] by using an IL-4-dependent pathway [56]. This modulation results in a lack of fibrotic capsule development or low thickness around the Dox-Si-M. Madden et al., (2010) [57] obtained a minimum fibrosis and a maximum vascularization combined with the cellular increase of macrophages of the M2 phenotype with fabricated poly (2-hydroxyethyl methacrylate-comethacrylic acid) (pHEMA-co-MAA) hydrogel scaffolds. Changes in the ratio M1/M2 or an increase in the number of M2 macrophages can be a potential strategy to protect the biomaterial [58]. In consideration of the obtained results, it is speculated that specimens treated with membranes that showed higher M1/M2 ratio (Dox-Si-Ms at the top regions of the defect) (Figure 4) may follow a chronic pro-inflammatory tissue reaction, leading to immediate adverse consequences for tissue remodeling, such as fibrous encapsulation, as stated previously [59]. The only time point of six weeks practiced in the present research may be considered as a shortcoming of our protocol. Therefore, a more extended time, in future research, would help to understand the present results in more depth.

The present research has analyzed the bone reconstruction process through the principal cell populations involved in the three main sequential phases of bone regeneration. It comprises inflammation, repair and remodeling, where osteoblasts, osteocytes, osteoclasts, macrophages and angiogenesis participate in concert [17]. The membranes studied that cover the bone defect promote angiogenesis with vascular vessels being more present near the membrane, as well as M1 and M2 macrophages that were confirmed to initiate the recruitment of vascular progenitor cells engaged in angiogenesis. In the vicinity of the membrane, a greater presence of pro-inflammatory M1, destined to bone repair, was detected. The relationship between the pro-inflammatory M1 and pro-regenerative M2 attained the highest values when the nanostructured membranes were doped with doxycycline that could lead to a chronic pro-inflammatory tissue reaction and fibrous encapsulation. In general, both osteoblasts and osteocytes grew closer to the membrane doped or not with zinc, where osteoblast-like cell activity and bioactivity were evidenced. Nevertheless, osteoclasts appeared uniformly distributed through the bone defect close to some multinucleated giant cells and M1 macrophages. For future research, a challenging strategy could be to determine if these membranes destined to guided bone regeneration might perform as a real scaffold, and therefore to analyze the interstitial growth of these membrane-recruited cells and vascular vessels within their electro-spinned nanostructure.

## 5. Conclusions

The presence of doped electrospinned membranes covering the critical size bone defects performed in experimental animals promoted, preferentially, the development of vascular vessels and all cell populations in the top regions of the bone defect compared to the bottom ones. In the vicinity of the membrane, a prominent angiogenic, osteoconductive and osteoinductive environment was triggered. Blood vessel count grew exponentially in the presence of the membranes, especially in the top regions and along the entire length of the membrane, forming a capillary network from side to side. Macrophages, osteoblasts and osteocytes increased in the top regions of the bone defects, close to the membrane; meanwhile, osteoclasts were uniformly distributed through the defect, whose counting was lower than that of osteoblasts in both top/bottom and lateral/central regions of the defect. Zn-doped membranes determined a major presence of osteocytes, osteoblasts and vascular vessels in the top regions of the defect. Dox-doped membranes contributed to an increase in both M1 and M2 macrophages, and so the M1/M2 ratio in the top regions, prone to fibrous encapsulation. The microenvironment that regulates and coordinates direct cell-to-cell contact or crosstalk among macrophages, osteoblasts, osteoclasts, osteocytes and vascular vessels was observed in the present histological sections. The promoted angiogenic, osteoconductive and osteoinductive environment would mean an improvement in the clinical process of human bone regeneration. Nevertheless, before extrapolating these results to humans, complementary investigations with large animals should be performed.

## Figures and Tables

**Table 1:** (A) Mean ± Standard Error of M1 and M2 macrophage counts in the selected study section. The M1/M2 ratio was also calculated for each studied membrane. (B) Statistical results (*p* values) after pairwise comparisons between different membranes. (C) Mean ± Standard Error of M1/M2 macrophage ratio when comparing top (R1, R2, R3) and bottom (R4, R5, R6) regions at the selected study section. (D) Mean ± Standard Error of M1/M2 macrophage ratio when comparing lateral (R1, R3, R4, R6) and central (R2, R5) regions at the selected study section. Bold numbers mean significance at *p* < 0.05.

A			
	M1	M2	M1/M2
Si-M	17.23 ± 2.05	5.57 ± 0.75	4.38 ± 0.69
Zn-Si-M	13.15 ± 1.56	5.30 ± 0.71	3.67 ± 0.71
Dox-Si-M	16.70 ± 1.75	7.19 ± 0.96	4.07 ± 0.63
Sham	18.41 ± 2.33	7.51 ± 1.13	2.42 ± 0.28
**B**			
	M1	M2	M1/M2
Si-M vs. Zn-Si-M	0.08	0.45	0.63
Si-M vs. Dox-Si-M	0.78	0.54	0.71
Si-M vs. Sham	0.68	0.39	0.27
Zn-Si-M vs. Dox-Si-M	0.34	0.47	0.85
Zn-Si-M vs. Sham	0.31	0.10	0.37
Dox-Si-M vs. Sham	0.64	0.92	0.51
**C**			
	M1/M2 TOP	M1/M2 BOTTOM	*p*-value pairwise
Si-M	5.12 ± 0.93	3.63 ± 1.02	0.69
Zn-Si-M	4.16 ± 1.24	3.17 ± 0.69	0.57
Dox-Si-M	6.08 ± 1.18	2.06 ± 0.31	**0.00**
Sham	2.23 ± 0.33	2.60 ± 0.45	0.55
*p*-value ANOVA	**0.04**	0.35	**0.03**
**D**			
	M1/M2 LATERAL	M1/M2 CENTRAL	*p*-value pairwise
Si-M	3.94 ± 0.63	5.26 ± 1.64	0.51
Zn-Si-M	4.61 ± 1.04	1.79 ± 0.31	0.41
Dox-Si-M	4.15 ± 0.78	3.91 ± 1.09	0.33
Sham	2.78 ± 0.40	1.69 ± 0.23	**0.05**
*p*-value ANOVA	0.360	**0.031**	**0.03**

## Data Availability

The data presented in this study are available on request from the corresponding author.

## Supplementary Materials

The following supporting information can be downloaded at: https://www.mdpi.com/article/10.3390/polym15071726/s1, Figure S1: Two bone defects were practiced on each side of the skull midline (A), separated 3 mm from each other (B, C). Defects dimensions were 8 mm of diameter and 3 mm of depth. They were prepared using a trephine (Helmut-Zepf Medical Gmbh, Seitingen, Germany) coupled to an implant micromotor operating at 2000 rpm under saline irrigation. The trephine presented an external diameter of 8 mm, a length of 30 mm, and teeth of 2.35 mm; Figure S2: The critical defects permitted the observation of the dura mater (arrow). A membrane is being positioned on a calvarial defect (asterisk). Three membranes were placed. The sham group may also be observed; Figure S3: Sutures were made, using resorbable material, on the following sequences: Periosteal (4/0) (A), sub-epidermal (4/0) (B), and skin (2/0) (C) making simple stitches as close as possible to the edge; Figure S4: (A) Bone histology images observed at R3 section obtained after using a Dox-Si-M, by coloration with toluidine blue to visualize endothelial cells (arrows) and endothelial tissues (pointers) that proliferated within the bone defect. The dotted square indicates Figure 4B, magnified. (B) Multinucleated giant cells (arrow), M1 macrophages with visible lamellipodia (pointers), and an osteoclast (arrowhead) are observed in the vicinity of the membrane toward the bone defect, mixed with fibrous and connective tissue (asterisks); Figure S5: Bone histology images obtained after using Zn-Si-M at R1 regions, by dye with toluidine blue to visualize blood vessels, at six weeks of healing time. Single arrows point the presence of blood vessels. Bone cells are in close contact with marrow elements and the contiguous vasculature. Osteocyte with its mineral lacuna (double arrow), osteoblast (faced arrow), macrophages (asterisks), and osteoclast (pointer) may be observed; Figure S6: Bone histology images observed at R3 (A) and R6 (B) sections obtained after using Zn-Si-Ms, and at R1 section when Si-M was used (C) by coloration with toluidine blue to visualize osteoblasts and other bone cells, at 6 weeks of healing time. Single arrows point the presence of aligned osteoblasts, with typical cuboid shape. Some polarized osteoblast show part of their cell membrane in direct contact with the bone surface, unveiling many cytoplasmic processes, achieving the newly deposited osteoid (OS). Pointers are indicative of osteoclasts. Osteoclasts and osteoblasts connect with each other through direct cell-cell contact and extracellular matrix interaction (dotted circles in S6B, S6C). Faced arrows mean canaliculi, and asterisks mark osteocytes and their filapodia (S6B). Lining cells, in close contact with osteoclasts, are signaled by arrowheads in “S6A” and “S6B”; Figure S7: Bone histology images obtained after analyzing the R5 sections in samples treated with Dox-Si-Ms (A and B), by dye with toluidine blue to visualize macrophages at the bone defect, at 6 weeks of healing time. M1 (pointers) and M2 (single arrows) macrophages were observable. Big (double arrows) and small blood vessels (faced arrows) are also shown; Figure S8: Bone histology obtained after using Si-Ms observed at R1 (A, B) and R5 (C) sections dyed with toluidine blue to visualize osteocytes, at 6 weeks of healing time. Single arrows point the presence of osteocytes. Canaliculi crossed through the bone matrix to bridge up a large extend of the membranes (double arrows) (Figures S8A and S8B). The extended network of osteocyte dendritic processes which reproduced the characteristic dendritic morphology joined at gap junctions (pointers) (Figures S8A, S8B and S8C). They were connected and intersected with each other by fine cytoplasmic processes and their branches. An osteocyte appeared embedded within its mineral lacuna (asterisk) (Figure S8A); Figure S9: (A) Bone histology images obtained after analysing the R1 section when using Si-M at 6 weeks of healing time; M1 (pointers) and M2 (single arrows) macrophages were observable. Big (double arrows) and small blood vessels (faced arrows) are also shown. (B) Analysis at R3 when Zn-Si-M were employed, by dye with toluidine blue to visualize macrophages at the bone defect, at 6 weeks of healing time. M1 (pointers) and M2 (single arrows) macrophages were observable. Big (double arrows) and small blood vessels (faced arrows) are also shown. At high magnification (100x), a population of both M1 and M2 macrophages may be observed in “D”; Table S1: Mean ± Standard Error of blood vessels and bone cells counts at the selected study section when comparing; (A) the presence of membrane over the defect versus no membrane and (B) top (R1, R2, R3) and bottom (R4, R5, R6) regions. Statistical results (*p* values) after pairwise comparisons. Bold numbers mean significance at *p* < 0.05; Table S2: Mean ± Standard Error of blood vessels and bone cells counts at the selected study section when comparing (A) the presence of membrane over the defect versus no membrane and (B) lateral (R1, R3, R4, R6) and central (R2, R5) regions. Statistical results (*p* values) after pairwise comparisons. Bold numbers mean significance at *p* < 0.05; Table S3: Mean ± Standard Error of blood vessels and bone cells counts at the selected study section within each of the regions into which the defect is divided. Statistically significant differences are in bold *p* < 0.05 [60].

## Abbreviations

ATP	Adenosine Triphosphate
AFM	Atomic Force Microscopy (AFM)
BMP	Bone Morphogenetic Protein
CM	Collagen Membrane
Dox-Si-M	SiO2-NPs doped membrane functionalized with Dox
Dox	Doxycycline
FESEM	Field Emission Scanning Electron Microscopy
GBR	Guided Bone Regeneration
HCl	Hydrochloric Acid
HEA	Hydroxyethyl acrylate
HEMA	Hydroxyethyl methacrylate
IL-1	Interleukin 1
IL-10	Interleukin 10
IL-4	Interleukin 4
IL-6	Interleukin 6
M1	Macrophage phenotype 1
M2	Macrophage phenotype 2
MA	Methyl acrylate
MMA	Methyl methacrylate
MMP	Matrix Metalloprotease
pHEMA-co-MAA	poly (2-hydroxyethyl methacrylate-comethacrylic acid)
PTFE	Polytetrafluoroethylene
SD	Standard deviation
Si-M	SiO2-NPs doped membrane
SiO_2_-NPs	silicon oxide nanoparticles
TB	Toluidine Blue
TNF-α	Tumor Necrosis Factor-α
VEGF	Vascular Endothelial Grow Factor
Zn-Si-M	SiO_2_-NPs doped membrane functionalized with Zn
Zn	Zinc
