# Supplementary material for: Doped Electrospinned Material-Guides High Efficiency Regional Bone Regeneration
Source: Polymers (Basel). 2023 Mar 30;15(7):1726. doi: 10.3390/polym15071726 (PMC10097153; doi:10.3390/polym15071726)
Supplement: Supplementary file 1 [file polymers-15-01726-s001.zip › polymers-2271444-supplementary.pdf]

Supplementary material

Doped electrospinned material-guides high efficiency regional bone regeneration.

Manuel Toledano [1,2,†], Cristina Vallecillo [1,†], María-Angeles Serrera-Figallo [3], Marta Vallecillo-Rivas [1,*], Aida Gutierrez-Corrales [3], Christopher D. Lynch [4] and Manuel Toledano-Osorio [1].

1 Faculty of Dentistry, Colegio Máximo de Cartuja s/n, University of Granada, 18071 Granada, Spain
2 Biosanitary Research Institute, IBS, Granada, 18012, Spain
3 Faculty of Dentistry, Oral Surgery Section, University of Sevilla, Avicena s/n, 41009 Sevilla, Spain
4 Restorative Dentistry, University Dental School & Hospital, University College Cork, Wilton, Cork T12 E8YV, Ireland
* Correspondence: mvallecillo@correo.ugr.es (M.V.-R.).
† These authors contributed equally to this work.

Anex S1. Electrospinning technique

Electrospinning is defined as a versatile approach to create nanofibrous biomaterials that constitute scaffolds for tissue regeneration. These electrospun nanofiber matrices mimic the morphology and exhibit similar morphological characteristics to that of the extracellular matrix. In the last decades, the electrospinning technique has improved more than ever before being these scaffolds applied in in the regeneration of various soft tissues, including non-connective (vascular, muscle, and neural tissue) and connective (ligament, skin, and tendon) tissues [S1,S2].

The electrospinning technique consists of creating ultrafine fibers by applying an electrostatic field. With the application of a high potential, fibers can be produced with a diameter range from 3 nm to 10 µm, while also exhibiting a high porosity, surface area to volume ratio, and flexibility. Four components comprised an electrospinning apparatus: a source of high voltage (1–30 kV), a metallic needle or capillary, a grounded conductive collector (which can be a rotating drum or a flat plate), and a syringe pump. High potential (14–16 kV) is applied between the metal collector and the polymer solution droplet in the syringe. Due to the electrostatic force the drop turns into a hemispherical form and when the electrostatic potential difference is high enough to overcome the surface tension of the polymer drop located at the tip of the metal needle, an elongated cone is generated. This results in the production of a charged liquid jet that is subjected to beat instabilities causing disturbances that cause the jet to take on a longer and thinner shape due to plastic deformation, elongating its length thousands of times its original size. Plastic deformation tends to dry out the jet, solvents evaporate, and ultrafine fibers result [S2].

References:

Ref S1. *Tebyetekerwa, M.; Ramakrishna, S. What Is Next for Electrospinning? Matter 2020, 2, 279–283.*

Ref S2. *Toledano M, Toledano-Osorio M, Carrasco-Carmona Á, Vallecillo C, Toledano R, Medina-Castillo AL, Osorio R. State of the Art on Biomaterials for Soft Tissue Augmentation in the Oral Cavity. Part II: Synthetic Polymers-Based Biomaterials. Polymers (Basel). 2020 Aug 17;12(8):1845. doi: 10.3390/polym12081845. PMID: 32824577; PMCID: PMC7465038.*

Figure S1. Two bone defects were practiced on each side of the skull midline (A), separated 3 mm from each other (B, C). Defects dimensions were 8 mm of diameter and 3 mm of depth. They were prepared using a trephine (Helmut-Zepf Medical Gmbh, Seitingen, Germany) coupled to an implant micromotor operating at 2000 rpm under saline irrigation. The trephine presented an external diameter of 8 mm, a length of 30 mm, and teeth of 2.35 mm.

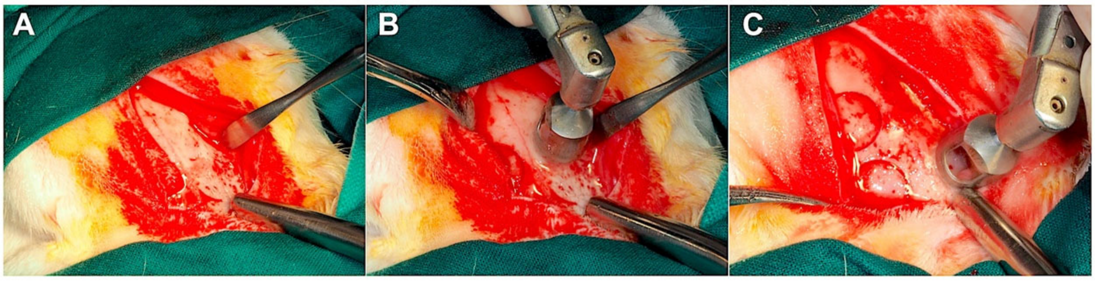

Figure S2. The critical defects permitted the observation of the *dura mater* (arrow). A membrane is being positioned on a calvarial defect (asterisk). Three membranes were placed. The sham group may also be observed.

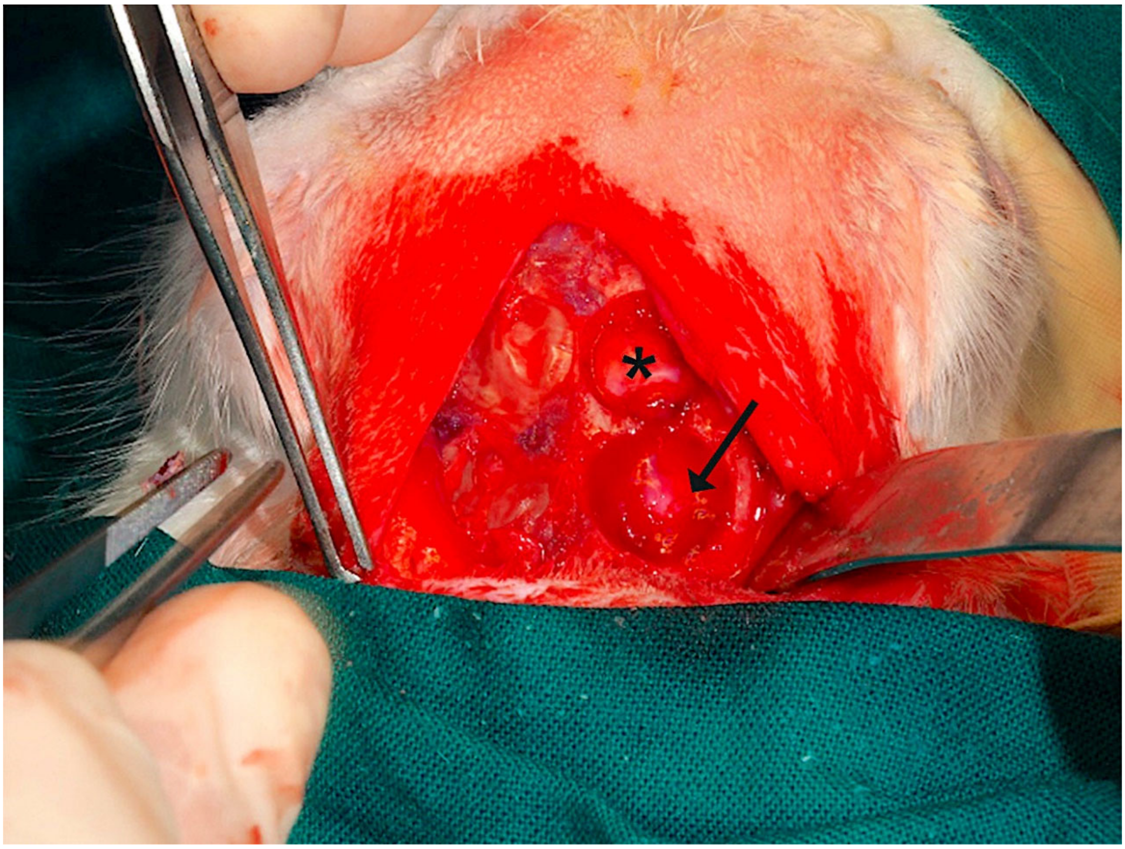

Figure S3. Sutures were made, using resorbable material, on the following sequences: Periosteal (4/0) (A), sub-epidermal (4/0) (B), and skin (2/0) (C) making simple stitches as close as possible to the edge.

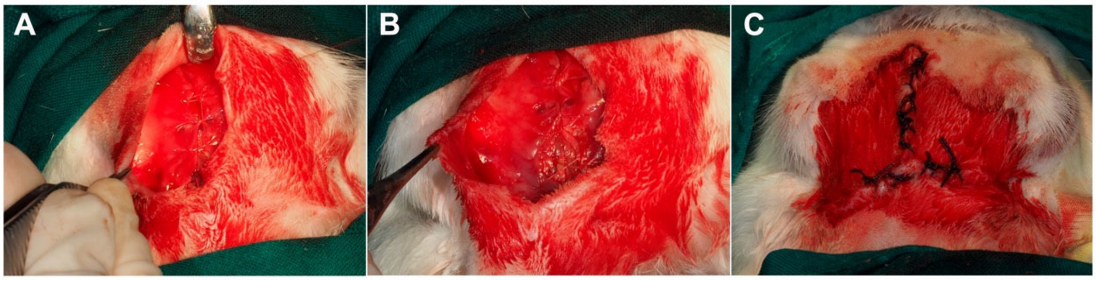

Figure S4: (**A**) Bone histology images observed at R3 section obtained after using a Dox-Si-M, by coloration with toluidine blue to visualize endothelial cells (arrows) and endothelial tissues (pointers) that proliferated within the bone defect. The dotted square indicates Figure 4B, magnified. (**B**) Multinucleated giant cells (arrow), M1 macrophages with visible lamellipodia (pointers), and an osteoclast (arrowhead) are observed in the vicinity of the membrane toward the bone defect, mixed with fibrous and connective tissue (asterisks).

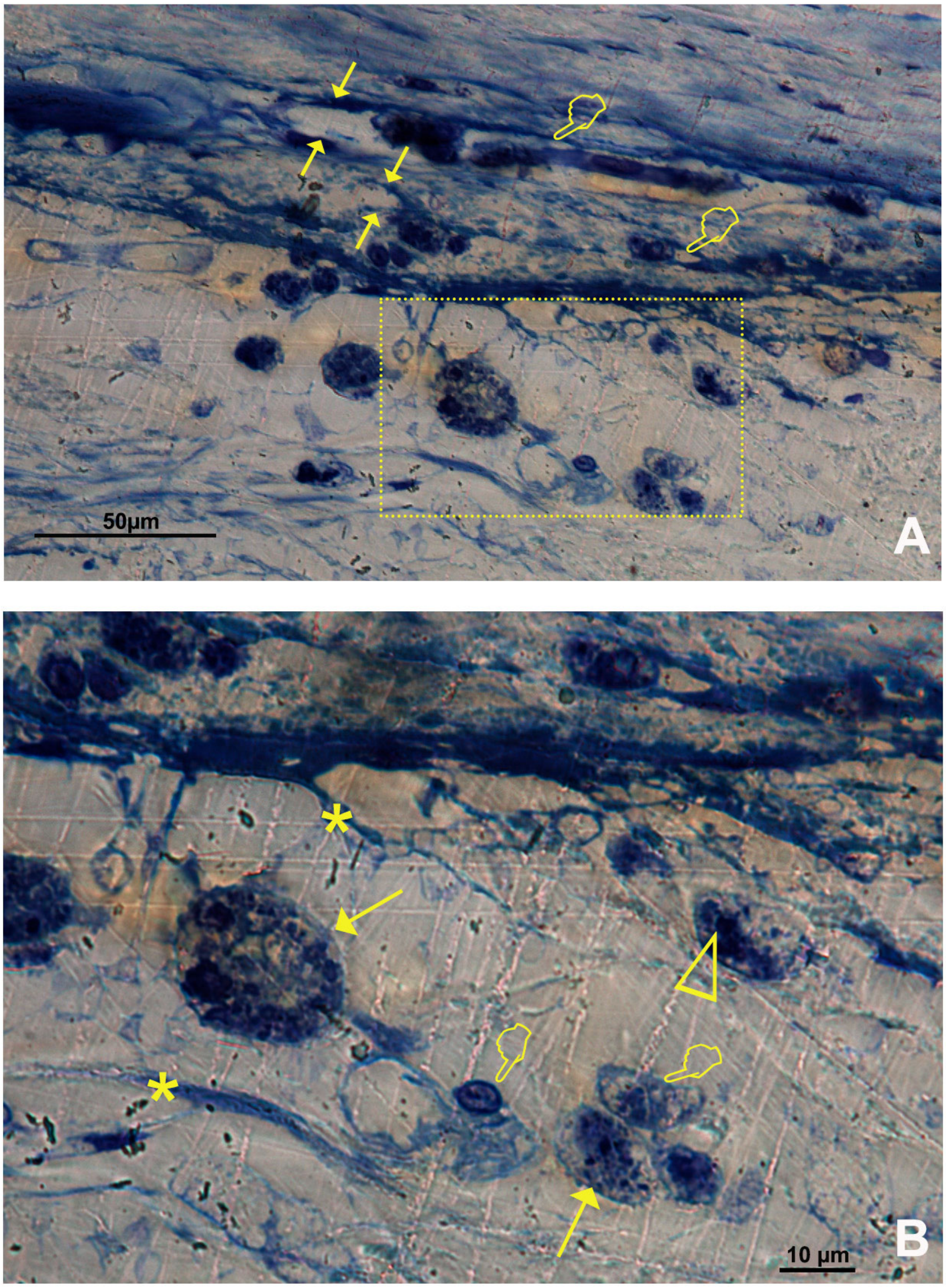

Figure S5: Bone histology images obtained after using Zn-Si-M at R1 regions, by dye with toluidine blue to visualize blood vessels, at six weeks of healing time. Single arrows point the presence of blood vessels. Bone cells are in close contact with marrow elements and the contiguous vasculature. Osteocyte with its mineral lacuna (double arrow), osteoblast (faced arrow), macrophages (asterisks), and osteoclast (pointer) may be observed.

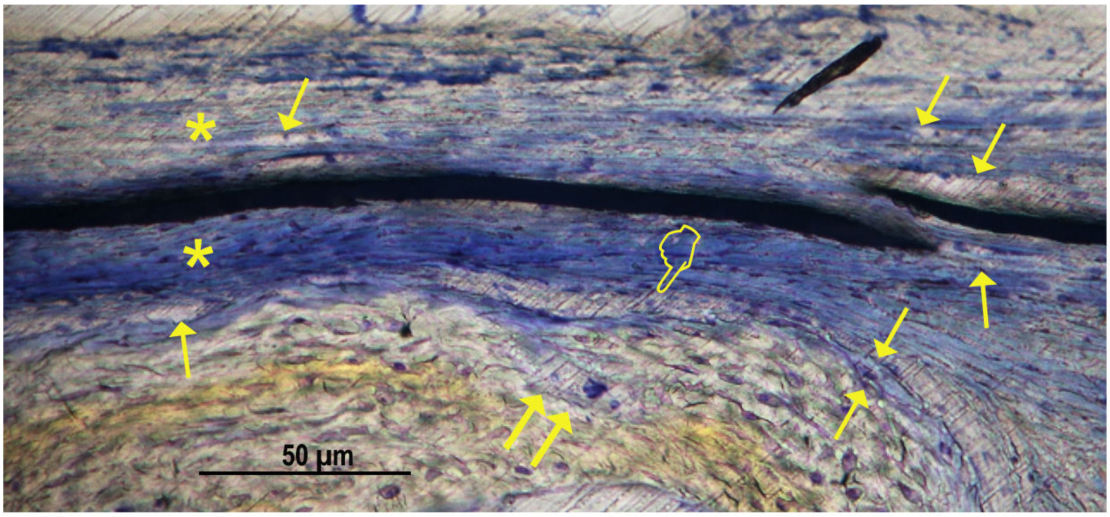

Figure S6: Bone histology images observed at R3 (**A**) and R6 (**B**) sections obtained after using Zn-Si-Ms, and at R1 section when Si-M was used (**C**) by coloration with toluidine blue to visualize osteoblasts and other bone cells, at 6 weeks of healing time. Single arrows point the presence of aligned osteoblasts, with typical cuboid shape. Some polarized osteoblast show part of their cell membrane in direct contact with the bone surface, unveiling many cytoplasmic processes, achieving the newly deposited osteoid (OS). Pointers are indicative of osteoclasts. Osteoclasts and osteoblasts connect with each other through direct cell-cell contact and extracellular matrix interaction (dotted circles in S6B, S6C). Faced arrows mean canaliculi, and asterisks mark osteocytes and their filapodia (S6B). Lining cells, in close contact with osteoclasts, are signaled by arrowheads in "S6A" and "S6B".

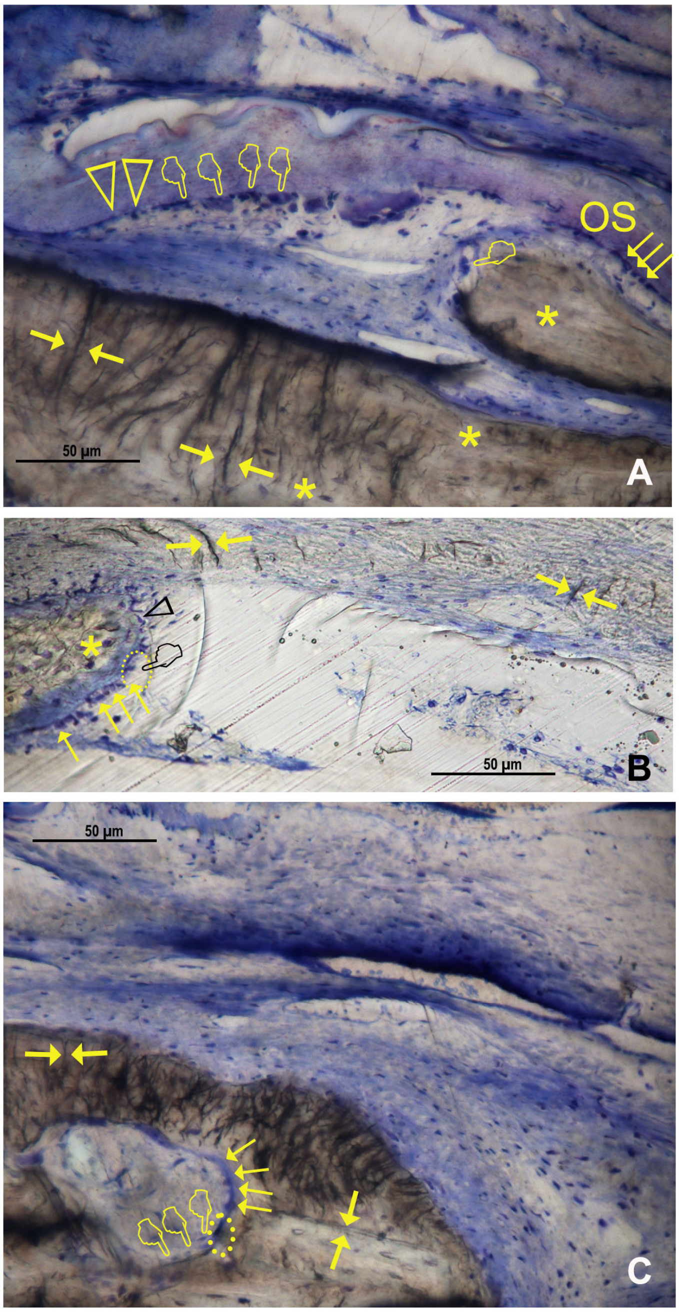

Figure S7: Bone histology images obtained after analyzing the R5 sections in samples treated with Dox-Si-Ms (**A** and **B**), by dye with toluidine blue to visualize macrophages at the bone defect, at 6 weeks of healing time. M1 (pointers) and M2 (single arrows) macrophages were observable. Big (double arrows) and small blood vessels (faced arrows) are also shown.

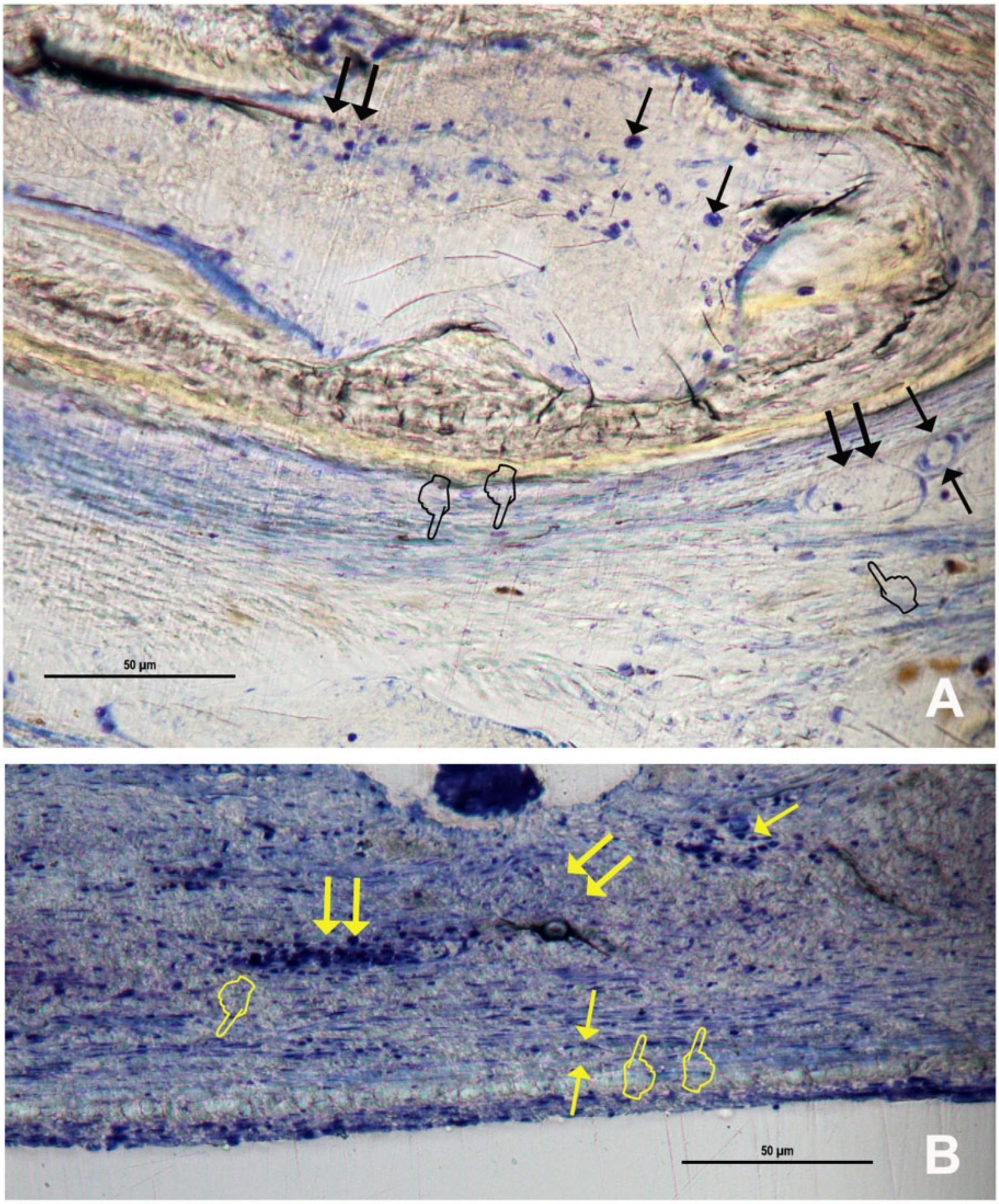

Figure S8: Bone histology obtained after using Si-Ms observed at R1 (**A**, **B**) and R5 (**C**) sections dyed with toluidine blue to visualize osteocytes, at 6 weeks of healing time. Single arrows point the presence of osteocytes. Canaliculi crossed through the bone matrix to bridge up a large extend of the membranes (double arrows) (Figures S8A, S8B). The extended network of osteocyte dendritic processes which reproduced the characteristic dendritic morphology joined at gap junctions (pointers) (Figures S8A, S8B, S8C). They were connected and intersected with each other by fine cytoplasmic processes and their branches. An osteocyte appeared embedded within its mineral lacuna (asterisk) (Figure S8A).

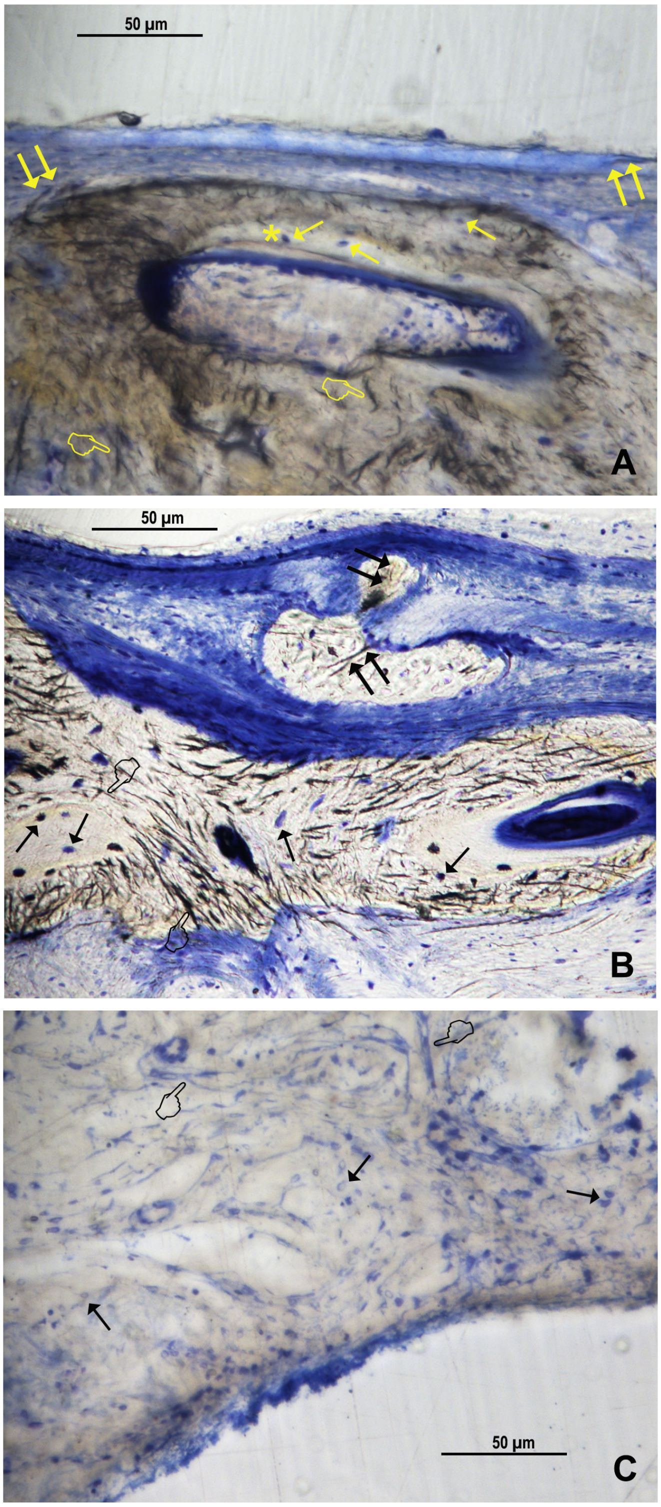

Figure S9: (**A**) Bone histology images obtained after analysing the R1 section when using Si-M at 6 weeks of healing time; M1 (pointers) and M2 (single arrows) macrophages were observable. Big (double arrows) and small blood vessels (faced arrows) are also shown. (**B**) Analysis at R3 when Zn-Si-M were employed, by dye with toluidine blue to visualize macrophages at the bone defect, at 6 weeks of healing time. M1 (pointers) and M2 (single arrows) macrophages were observable. Big (double arrows) and small blood vessels (faced arrows) are also shown. At high magnification (100x), a population of both M1 and M2 macrophages may be observed in "D".

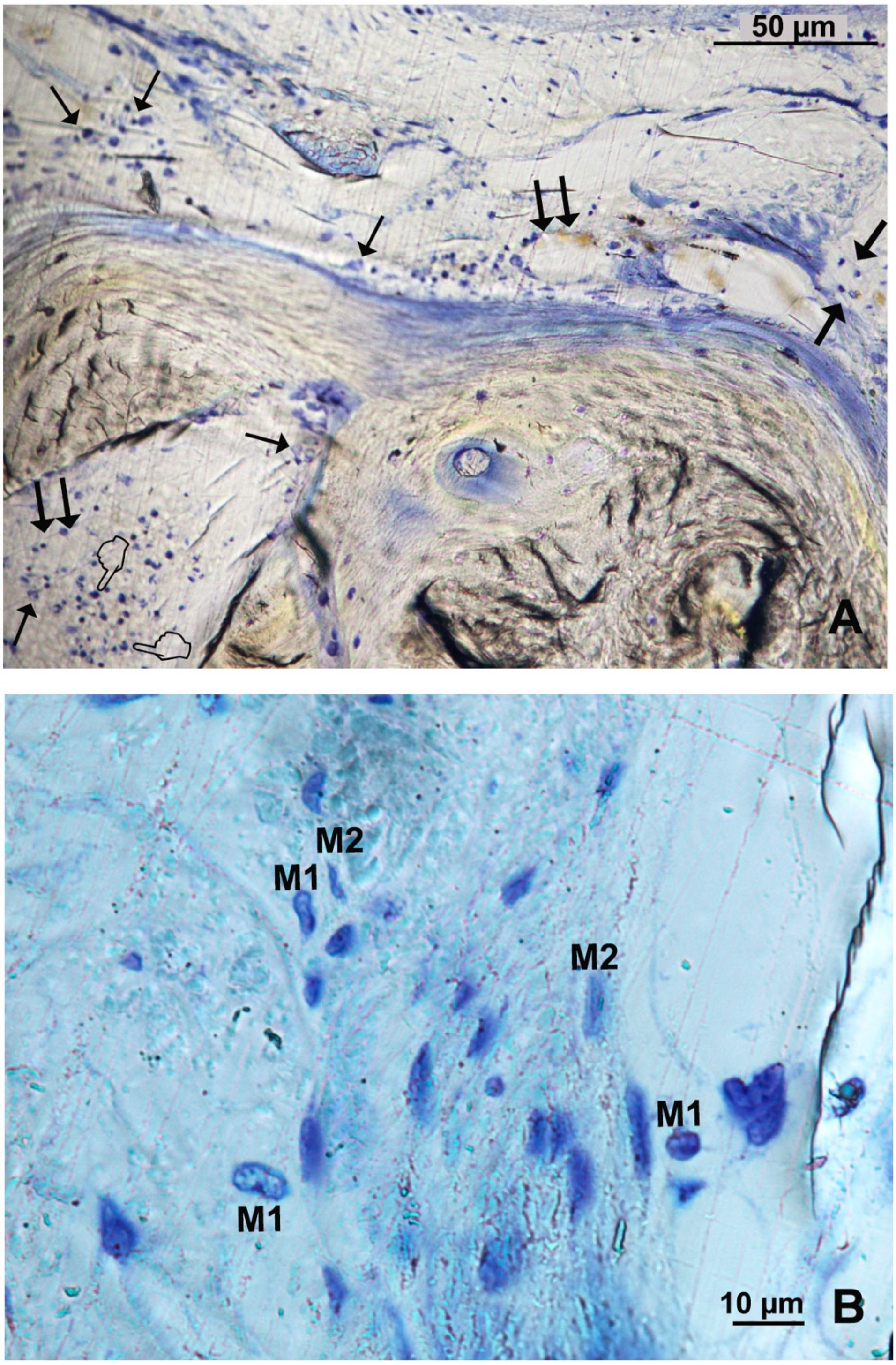

Table S1: Mean ± Standard Error of blood vessels and bone cells counts at the selected study section when comparing; (A) the presence of membrane over the defect versus no membrane and (B) top (R1, R2, R3) and bottom (R4, R5, R6) regions. Statistical results (p values) after pairwise comparisons. Bold numbers mean significance at $p < 0.05$.

			**A**			
**With membrane**	Blood Vessels	Osteoblasts	Osteocytes	Osteoclasts	M1	M2
Top	1.21 ± 0.14	9.99 ± 0.92	19.55 ± 1.94	0.66 ± 0.1	19.57 ± 1.56	7.62 ± 0.76
Bottom	0.55 ± 0.11	6.507 ± 0.82	13.71 ± 1.88	0.39 ± 0.1	11.80 ± 1.31	4.46 ± 0.55
p-value	**0.00**	**0.00**	**0.00**	**0.01**	**0.00**	**0.00**
**Without memabrane**	Blood Vessels	Osteoblasts	Osteocytes	Osteoclasts	M1	M2
Top	1.00 ± 0.27	2.49 ± 0.87	9.19 ± 2.10	0.28 ± 0.13	18.97 ± 3.42	8.86 ± 1.92
Bottom	0.88 ± 0.21	1.13 ± 0.37	3.26 ± 1.18	0.06 ± 0.04	17.86 ± 3.20	6.17 ± 1.18
p-value	0.85	0.12	**0.00**	0.09	0.52	0.09
			**B**			
**TOP** Membrane	Blood Vessels	Osteoblasts	Osteocytes	Osteoclasts	M1	M2
Si-M	1.27 ± 0.24	11.79 ± 1.72	23.26 ± 3.27	0.74 ± 0.19	19.24 ± 2.99	6.21 ± 1.12
Zn- Si-M	1.44 ± 0.30	10.24 ± 1.45	19.48 ± 3.39	0.77 ± 0.21	14.73 ± 2.26	6.65 ± 1.12
Dox- Si-M	0.93 ± 0.19	8.13 ± 1.62	16.21 ± 3.42	0.47 ± 0.14	24.53 ± 2.72	9.83 ± 1.60
Sham	1.00 ± 0.27	2.49 ± 0.87	9.19 ± 2.10	0.28 ± 0.13	18.97 ± 3.42	8.86 ± 1.92
**BOTTOM** Membrane	Blood Vessels	Osteoblasts	Osteocytes	Osteoclasts	M1	M2
Si-M	0.53 ± 0.18	7.58 ± 1.74	16.30 ± 3.47	0.48 ± 0.24	15.21 ± 2.78	4.92 ± 1.01
Zn- Si-M	0.67 ± 0.22	6.70 ± 1.36	12.04 ± 2.93	0.41 ± 0.17	11.58 ± 2.14	3.94 ± 0.87
Dox- Si-M	0.46 ± 0.17	5.35 ± 1.18	12.92 ± 3.36	0.28 ± 0.11	8.88 ± 1.80	4.54 ± 1.00
Sham	0.88 ± 0.21	1.13 ± 0.37	3.26 ± 1.18	0.06 ± 0.04	17.86 ± 3.20	6.17 ± 1.18
p-value **Top vs. Bottom**	Blood Vessels	Osteoblasts	Osteocytes	Osteoclasts	M1	M2
Si-M	**<0.01**	**<0.01**	0.06	0.08	**0.04**	0.36
Zn- Si-M	**0.04**	**0.03**	**0.05**	0.16	0.38	0.07
Dox- Si-M	**0.02**	0.17	0.12	0.30	**<0.01**	**<0.01**
Sham	0.85	0.12	**<0.01**	0.09	0.52	0.09

Table S2: Mean ± Standard Error of blood vessels and bone cells counts at the selected study section when comparing (A) the presence of membrane over the defect versus no membrane and (B) lateral (R1, R3, R4, R6) and central (R2, R5) regions. Statistical results (p values) after pairwise comparisons. Bold numbers mean significance at $p < 0.05$.

			**A**			
**With membrane**	Blood Vessels	Osteoblasts	Osteocytes	Osteoclasts	M1	M2
Lateral	0.90 ± 0.11	9.57 ± 0.83	19.12 ± 1.82	0.56 ± 0.09	16.16 ± 1.28	6.08 ± 0.59
Central	0.83 ± 0.16	5.62 ± 0.84	11.63 ± 1.78	0.44 ± 0.13	16.16 ± 1.75	5.96 ± 0.80
p-value	0.88	0.47	0.42	0.52	0.54	0.09
**Without membrane**	Blood Vessels	Osteoblasts	Osteocytes	Osteoclasts	M1	M2
Lateral	1.01 ± 0.21	1.98 ± 0.66	6.81 ± 1.57	0.21 ± 0.09	21.12 ± 3.03	8.01 ± 1.25
Central	0.80 ± 0.30	1.48 ± 0.53	5.07 ± 1.93	0.09 ± 0.09	13.00 ± 3.41	6.52 ± 2.28
p-value	0.50	0.97	0.53	0.35	**0.01**	0.08
			**B**			
**LATERAL** Membrane	Blood Vessels	Osteoblasts	Osteocytes	Osteoclasts	M1	M2
Si-M	0.89 ± 0.17	10.81 ± 1.62	22.32 ± 3.12	0.55 ± 0.13	19.66 ± 2.79	6.36 ± 1.01
Zn- Si-M	1.14 ± 0.23	9.61 ± 1.34	17.52 ± 3.00	0.68 ± 0.19	14.12 ± 1.84	4.92 ± 0.74
Dox- Si-M	0.69 ± 0.16	8.40 ± 1.34	17.73 ± 3.29	0.46 ± 0.12	14.92 ± 1.98	6.93 ± 1.25
Sham	1.01 ± 0.21	1.98 ± 0.66	6.81 ± 1.57	0.21 ± 0.09	21.12 ± 3.03	8.01 ± 1.25
**CENTRAL** Membrane	Blood Vessels	Osteoblasts	Osteocytes	Osteoclasts	M1	M2
Si-M	0.93 ± 0.30	7.43 ± 1.73	14.70 ± 3.48	0.75 ± 0.38	12.36 ± 2.43	3.98 ± 0.98
Zn- Si-M	0.87 ± 0.33	6.17 ± 1.30	12.24 ± 3.10	0.39 ± 0.14	11.22 ± 2.90	6.04 ± 1.57
Dox- Si-M	0.71 ± 0.21	3.42 ± 1.28	8.23 ± 2.68	0.21 ± 0.11	20.27 ± 3.44	7.71 ± 1.48
Sham	0.80 ± 0.30	1.48 ± 0.53	5.07 ± 1.93	0.09 ± 0.09	13.00 ± 3.41	6.52 ± 2.28
p-value **Lateral vs. Central**	Blood Vessels	Osteoblasts	Osteocytes	Osteoclasts	M1	M2
Si-M	0.91	0.30	0.23	0.64	0.76	0.85
Zn- Si-M	0.81	0.55	0.99	0.64	0.26	0.79
Dox- Si-M	0.96	0.40	0.77	0.91	0.09	**0.01**
Sham	0.50	0.97	0.53	0.35	**0.01**	0.08

Table S3: Mean ± Standard Error of blood vessels and bone cells counts at the selected study section within each of the regions into which the defect is divided. Statistically significant differences are in bold $p < 0.05$.

**R1**	Blood Vessels	Osteoblasts	Osteocytes	Osteoclasts	M1	M2
Si-M	1.77 ± 0.45	14.45 ± 3.31	29.95 ± 6.70	1.14 ± 0.40	28.91 ± 7.32	9.23 ± 2.63
Zn- Si-M	1.39 ± 0.51	8.91 ± 2.36	21.61 ± 5.40	0.48 ± 0.21	7.22 ± 2.02	4.61 ± 1.60
Dox- Si-M	1.00 ± 0.34	10.92 ± 3.20	18.38 ± 6.09	0.63 ± 0.25	27.04 ± 5.02	13.46 ± 3.70
Sham	1.77 ± 0.39	1.48 ± 0.93	7.17 ± 2.95	0.26 ± 0.18	16.65 ± 4.70	7.70 ± 2.02
*p-v*alue	0.61	**0.01**	**0.04**	0.14	**0.01**	0.12
**R2**	Blood Vessels	Osteoblasts	Osteocytes	Osteoclasts	M1	M2
Si-M	0.91 ± 0.41	11.64 ± 2.97	20.86 ± 4.90	0.64 ± 0.34	13.23 ± 3.38	4.32 ± 1.47
Zn- Si-M	1.61 ± 0.61	7.61 ± 1.55	17.04 ± 5.20	0.52 ± 0.21	16.43 ± 4.44	8.48 ± 2.45
Dox- Si-M	0.92 ± 0.36	3.71 ± 2.20	8.71 ± 4.43	0.25 ± 0.18	28.92 ± 5.22	9.88 ± 2.13
Sham	0.61 ± 0.43	1.87 ± 0.87	7.74 ± 3.36	0.17 ± 0.17	14.43 ± 5.31	8.52 ± 4.15
*p-v*alue	0.86	**0.01**	0.13	0.45	0.07	0.52
**R3**	Blood Vessels	Osteoblasts	Osteocytes	Osteoclasts	M1	M2
Si-M	1.14 ± 0.36	9.27 ± 2.64	18.95 ± 5.14	0.45 ± 0.23	15.59 ± 3.43	5.09 ± 1.37
Zn- Si-M	1.31 ± 0.43	14.17 ± 3.21	19.78 ± 6.94	1.30 ± 0.54	20.52 ± 4.42	6.87 ± 1.64
Dox- Si-M	0.88 ± 0.30	9.75 ± 2.80	21.54 ± 6.90	0.54 ± 0.28	17.63 ± 3.62	6.17 ± 2.06
Sham	1.22 ± 0.57	4.13 ± 2.29	12.65 ± 4.49	0.39 ± 0.29	25.83 ± 7.42	10.35 ± 3.57
*p-v*alue	0.90	0.09	0.74	0.24	0.51	0.42
**R4**	Blood Vessels	Osteoblasts	Osteocytes	Osteoclasts	M1	M2
Si-M	0.45 ± 0.23	14.55 ± 4.38	24.68 ± 7.05	0.27 ± 0.16	20.14 ± 6.78	6.18 ± 2.30
Zn- Si-M	0.83 ± 0.38	8.09 ± 2.67	17.83 ± 7.21	0.39 ± 0.20	15.57 ± 3.69	4.09 ± 1.47
Dox- Si-M	0.71 ± 0.44	6.17 ± 2.39	13.13 ± 6.45	0.13 ± 0.13	3.96 ± 1.39	3.42 ± 1.63
Sham	0.43 ± 0.23	0.83 ± 0.48	2.26 ± 1.32	0.04 ± 0.04	16.48 ± 4.61	4.48 ± 1.31
*p-v*alue	0.81	**0.01**	0.07	0.33	0.06	0.70
**R5**	Blood Vessels	Osteoblasts	Osteocytes	Osteoclasts	M1	M2
Si-M	0.95 ± 0.46	3.23 ± 1.34	8.55 ± 4.58	0.86 ± 0.69	11.50 ± 3.58	3.64 ± 1.34
Zn- Si-M	0.13 ± 0.13	4.74 ± 2.07	7.43 ± 3.19	0.26 ± 0.18	6.00 ± 3.51	3.61 ± 1.87
Dox- Si-M	0.50 ± 0.23	3.13 ± 1.37	7.75 ± 3.12	0.17 ± 0.12	11.63 ± 3.82	5.54 ± 2.01
Sham	1.00 ± 0.42	1.09 ± 0.63	2.39 ± 1.83	0.00 ± 0.00	11.57 ± 4.40	4.52 ± 1.94
*p-v*alue	0.21	0.36	0.54	0.33	0.67	0.86
**R6**	Blood Vessels	Osteoblasts	Osteocytes	Osteoclasts	M1	M2
Si-M	0.18 ± 0.14	4.95 ± 1.87	15.68 ± 2.40	0.32 ± 0.19	14.00 ± 3.37	4.95 ± 1.50
Zn- Si-M	1.04 ± 0.51	7.26 ± 2.33	10.87 ± 3.88	0.57 ± 0.45	13.17 ± 3.79	4.13 ± 1.15
Dox- Si-M	0.17 ± 0.10	6.75 ± 2.24	17.88 ± 7.14	0.54 ± 0.29	11.04 ± 3.44	4.67 ± 1.58
Sham	1.22 ± 0.42	1.48 ± 0.79	5.13 ± 2.74	0.13 ± 0.95	25.52 ± 7.06	9.52 ± 2.56
*p-v*alue	0.05	0.14	0.33	0.68	0.13	0.13
